# Differentiating Social and Moral Norms in Perceived Internalization

**DOI:** 10.3390/bs16050774

**Published:** 2026-05-14

**Authors:** Paul Deutchman, Fiona Y. Yang

**Affiliations:** 1 Philosophy, Politics, & Economics, Center for Social Norms and Behavioral Dynamics, University of Pennsylvania, Philadelphia, PA 19104, USA; 2 Master of Behavioral and Decision Sciences, University of Pennsylvania, Philadelphia, PA 19104, USA; yujin.yang@chicagobooth.edu; 3 Booth School of Business, University of Chicago, Chicago, IL 60637, USA

**Keywords:** social cognition, moral norms, social norms, fairness, harm, internalization

## Abstract

Why are some behaviors perceived as moral norms while others are perceived as social norms? In a preregistered study (N = 535), we examined how people perceive different types of moral behaviors and whether those perceptions help distinguish between moral and social norms. To test this, we assigned participants to one of five types of commonly studied norms—conventional, fairness, harm, generosity, and purity—and presented them with eight behaviors, four prescriptive and four proscriptive. To capture differences in belief internalization, participants answered a series of measures assessing their intrinsic motivation to follow the behaviors, beliefs about the importance of adhering to (or avoiding) the behaviors, and sensitivity to reputational concerns in their intentions to engage in them. We had two main findings: First, our measures of internalization varied across behavioral domains, such that harm behaviors were generally perceived as the most internalized and conventional behaviors as the least. Second, harm perceptions partially mediated differences in intrinsic motivation between harm and several other behavioral domains, suggesting that harm perceptions may underlie differences in internalization between social and moral norms. Together, our results reveal important differences in how people perceive social and moral norms, informing our understanding of norm cognition and internalization.

## 1. Introduction

### 1.1. Social and Moral Norms

Social norms are the tacit rules that guide and constrain behavior, influencing everything from how we divide and share resources to the food we eat for breakfast. Norms are pervasive in social life and play a critical role in structuring our behavior and society (Fehr & Fischbacher, 2004; Kelly & Davis, 2018; McDonald & Crandall, 2015). Yet despite their importance, the cognitive processes through which social norms influence beliefs and behavior remain poorly understood. One particularly important yet unresolved question in norm cognition concerns the relationship between social norms and moral norms.

Moral norms are closely related to social norms but are defined inconsistently across the literature. In some work, moral norms refer to social norms with high moral relevance (Berneri et al., 2024; Kroneberg et al., 2010; Meng et al., 2022) or in specific moral domains (like purity and fairness; Dungan et al., 2017; FeldmanHall et al., 2018), while other research treats them as synonymous with social norms (Lindström et al., 2018; Mulder, 2018; Smith & Masser, 2012). Consequently, it remains unclear whether social and moral norms actually correspond to the same underlying normative information or whether moral norms are meaningfully distinct from social norms. Answering this question will clarify the cognitive processes underlying norm cognition and may inform applied work using norm-based messaging interventions. For example, if people perceive moral norms as more inalterable and more important to follow than social norms—as previous work suggests (Sadek, 2024; Smetana, 2013; Smetana & Yoo, 2022)—then they may also be more likely to comply with and enforce those norms. Additionally, it remains unclear which types of moral behaviors most closely correspond to moral versus social norms. While harm norms are widely regarded as prototypical moral norms (Sadek, 2024; Gray et al., 2012) and conventional norms as prototypical social norms (Bicchieri, 2006; Smetana, 2013), fairness represents a more ambiguous case. Although fairness plays a key role in moral judgment (Baumard et al., 2013; Rai & Fiske, 2011; Graham et al., 2011; Tomasello, 2018) and is commonly regarded as a moral norm (Elster, 2006; FeldmanHall et al., 2018; Lindström et al., 2018), recent work suggests that fairness norms may be perceived more akin to conventional social norms than moral harm norms (Deutchman et al., 2024; Yucel et al., 2022; Yucel & Vaish, 2025). Consequently, the broad goal of the present work was to clarify the conceptual overlap between social and moral norms, as well as to formalize the conceptual definitions between these two types of normative information.

### 1.2. Are Moral Norms Distinct from Social Norms?

While prior work suggests that social and moral norms are closely related and overlapping concepts (Van Schoelandt, 2018), several prominent theories of norm cognition argue that social norms are categorically distinct from moral norms in several key respects. Social domain theory (SDT) distinguishes between two types of norm information: social conventions (i.e., conventional norms) and moral norms (Smetana, 2013; Smetana & Yoo, 2022). In this framework, social conventions—such as dress codes or table manners—are perceived as context-dependent, alterable, and relatively arbitrary, whereas moral norms, particularly those concerning harm, are viewed as more inalterable, less contextually relative, and more deserving of punishment when violated (Smetana, 2013; Smetana & Yoo, 2022). Thus, SDT suggests a clear distinction between social conventional and moral norms which emerges early in development. Importantly, however, SDT broadly conceptualizes both conventional and moral norms as subtypes of social norms. This conceptualization notably differs from other theories that posit a categorical distinction between social norms and moral norms.^1^

One influential theory that argues for a clear distinction between social and moral norms is Bicchieri’s social conditionality theory (SCT; 2006, 2016). In SCT, social norms are defined as socially conditional preferences: individuals comply with them to the extent that they believe others commonly engage in the behavior (*empirical* beliefs) and expect them to do so (*normative* beliefs). Behaviors that are not socially conditional—e.g., behaviors that individuals would follow regardless of others’ behavior or expectations—do not qualify as social norms in this framework. Moral norms, by contrast, constitute a distinct category of behavior that is not socially conditional; individuals are intrinsically motivated to follow them regardless of others’ behavior or expectations. Accordingly, SCT characterizes social norms and moral norms as distinct and mutually exclusive forms of normative motivation.

Together, this work suggests a fundamental difference between social and moral norms. Social norms are more flexible and alterable *because* they are socially constructed and externally motivated behaviors that depend on others’ beliefs and behavior. Moral norms, on the other hand, are less flexible and more objective, reflecting behaviors that individuals are motivated to follow regardless of their empirical or normative expectations. Consequently, the main distinction between social and moral norms posited by these theories concerns the intrinsic motivation to engage in these behaviors. Whether individuals are relatively more intrinsically or extrinsically motivated to engage in a norm largely depends on the extent to which we have internalized that behavior.

Internalization is the process by which norms become tied to an individual’s sense of self and social identity, such that compliance is intrinsically motivated rather than driven by external expectations or sanctions (Gavrilets & Richerson, 2017; Kelly, 2020). When norms are internalized, individuals intrinsically value them as ends in themselves, such that violations of these norms elicit psychological discomfort, including feelings of guilt or shame (Gavrilets & Richerson, 2017; Sullins et al., 2024). By contrast, behaviors motivated primarily by external incentives—such as rewards, punishment, or reputational concerns—are not considered internalized. Internalization is widely viewed as a core feature of our shared normative psychology and a growing body of evidence suggests that this ability may emerge early in development, possibly as young as 18 months of age (Essler et al., 2023; Schmidt et al., 2019). Consequently, internalization is thought to reflect an evolutionary adaptation that promotes cooperation and norm compliance by reducing the costs associated with information gathering, decision-making, and behavioral monitoring (Henrich & Ensminger, 2014; Gintis, 2003; Gavrilets & Richerson, 2017).

Internalization is frequently invoked as a key criterion for distinguishing moral norms from social norms. Across several theories of norm cognition (e.g., SCT, SDT), moral norms are characterized as internalized beliefs—beliefs individuals endorse and follow regardless of others’ behavior or expectations—whereas social norms are thought to rely more heavily on extrinsic motivations, such as social approval or reputational concerns (Bicchieri, 2006). Despite the centrality of internalization to these accounts, substantial theoretical ambiguity remains regarding what it means for a norm to be internalized and whether different types of normative content are internalized to different degrees.

In this paper, we argue that the relationship between internalization and norms is not as straightforward as “moral norms = internalized” and “social norms ≠ internalized”. For example, while a number of conventional social norms—such as saying “please” and “thank you”—are clearly internalized, there are very few moral norms that are *not* internalized. This suggests that, while not all internalized norms are moral norms, most moral norms are internalized. Put differently, internalization may be a necessary but not sufficient condition for a behavior to be categorized as a moral norm (but see Kelly & Davis, 2018, who argue that internalization is a key feature of all social norms). Importantly, we assume here that internalization exists on a continuum rather than as a discrete phenomenon (e.g., either internalized or not), such that behaviors can be relatively more or less internalized than others.

Moreover, the extent to which a norm is internalized may depend on its content or domain—in other words, not every norm is equally likely to be internalized. A growing body of work suggests that norms related to harm may be acquired and internalized more quickly and reliably than other moral norms, such as fairness. For example, developmental research indicates that harm norms emerge within the first two years of life, whereas fairness norms typically emerge later, around ages five to six (Blake et al., 2015; McAuliffe et al., 2017; House, 2018), while cross-cultural research indicates that harm norms are less culturally variable than fairness norms (Barrett et al., 2016; Yau & Smetana, 2003). Thus, one way of potentially distinguishing the social cognition of moral and social norms is to examine whether different moral domains vary systematically in their degree of internalization. If some behaviors commonly described as “moral” (e.g., generosity, fairness, harm, purity, etc.) are more readily internalized than others, this would be indicative of a hierarchy of moral norms such that some moral norms are “more moral” than others. Consequently, behaviors viewed as the most internalized should also be the most representative and prototypical examples of moral norms.

### 1.3. Differences Across Moral Domains

Moral Foundations Theory (MFT; Graham et al., 2011; Graham et al., 2013) is a nativist theory of morality that proposes that human morality is rooted in evolved adaptations to recurrent social problems (and thus differs from more constructivist accounts of morality which emphasize social and cultural learning; Paulus, 2020). MFT proposes that human morality comprises five (or, more recently, six; Atari et al., 2023) distinct domains and that differences between these domains can explain variation in moral values across individuals and groups (e.g., liberals and conservatives; Graham et al., 2009). The five original domains of MFT are harm–care, fairness–reciprocity, ingroup–loyalty, authority–respect, and purity–sanctity. MFT suggests there are important distinctions between how we perceive these different domains of morality, such that one could care highly about fairness and harm but little about authority and in-group loyalty. A large body of work in moral psychology has now investigated moral judgements according to this framework, finding mixed evidence to support the five foundation model (Iurino & Saucier, 2020). Nevertheless, while current evidence is not fully supportive of a five (or six; Harper & Rhodes, 2021) foundation model of morality, it does still suggest that there are important distinctions between types of norms commonly perceived as moral, such as between harm, fairness, and loyalty. Other theories of morality, such as the morality-as-cooperation theory (Curry et al., 2019; Curry, 2016), make similar claims by proposing multiple functionally distinct moral domains (such as kin altruism, reciprocity, bravery, etc.). Thus, while theorists disagree about the exact number and nature of moral domains, there is broad agreement that moral cognition is domain-specific and that individuals vary in the extent to which they value different moral domains.

Other recent work drawing on MFT supports the notion that there are distinct cognitive processes that underlie different moral domains. For example, several studies have identified a number of differences between the harm and purity domains: intent influences moral judgements of harm but not purity (Chakroff et al., 2016), mental state reasoning plays a larger role in moral judgements of harm than in purity violations (Dungan & Young, 2019), and harm and purity norms are relevant in distinct relational contexts (Dungan et al., 2017). Other work highlights systematic differences between harm and fairness, showing that both children and adults perceive fairness violations differently from harm violations (Yucel & Vaish, 2025; Yucel et al., 2022), and in some cases, perceive fairness as more similar to conventional norms (Deutchman et al., 2024; Yucel & Vaish, 2025). Together, a growing body of evidence suggests that there are important differences across several types of behaviors commonly conceptualized as moral norms. Examining whether these moral domains also differ in the extent to which they are internalized can therefore clarify whether different moral norms are supported by distinct cognitive processes and, more broadly, whether the distinction between moral and social norms is conceptually meaningful.

### 1.4. Present Study

This study investigated whether social and moral norms are psychologically distinct forms of normative information (Bicchieri, 2006; Smetana, 2013) or instead reflect a common underlying psychological construct (Van Schoelandt, 2018).^2^ To address this question, we randomly assigned participants to one of five behavioral domain conditions (conventional, harm, fairness, generosity, and purity). Namely, we compared conventional norms—widely regarded as prototypical social norms (Smetana et al., 2013; Smetana & Yoo, 2022)—and harm norms—widely regarded as the prototypical moral norm (Sadek, 2024; Gray et al., 2012)—with three other types of morally relevant behaviors often referred to as moral norms in the literature (fairness, generosity, purity; Chakroff et al., 2016; Deutchman et al., 2024; Dungan et al., 2017; Graham et al., 2011; Yucel & Vaish, 2025) across measures of belief internalization. Because internalization, as an internal psychological process, is difficult to directly measure, we operationalized it using three theoretically grounded indicators: intrinsic motivation to engage in the behavior (versus extrinsic motivation), perceived importance of adhering to prescriptive norms (or avoiding proscriptive norms), and sensitivity to reputational concerns in behavioral intentions. Greater intrinsic motivation and perceived importance, coupled with lower sensitivity to reputational concerns, were taken to indicate higher levels of internalization. Additionally, because prior work has found that prescriptive norms (i.e., behaviors we should engage in) and proscriptive norms (i.e., behaviors we should avoid) have distinct motivational underpinnings (Janoff-Bulman et al., 2009; Miles et al., 2025) and differentially influence behavioral intentions and moral attitudes (Pavey et al., 2018), we included both prescriptive and proscriptive norms for each behavioral domain.

Our predictions centered on whether perceived internalization differentiates moral norms from social norms. Namely, our primary research question was whether moral norms (e.g., harm) are perceived as psychologically distinct in measures of belief internalization from social norms (e.g., conventional norms). We predicted that harm norms would be perceived as the most internalized—e.g., most intrinsically motivated, most important to follow, and least sensitive to reputational concerns—as compared to the other domains. If harm norms are perceived as more strongly internalized, this would support theories positing a categorical distinction between moral and social norms (Bicchieri, 2006; Smetana, 2013). Alternatively, if harm norms do not differ from other normative behaviors along these internalization dimensions, this would call into question the view that moral norms constitute a distinct class of normative information. We further expected that conventional norms would be perceived as the least internalized, as reflected in lower intrinsic motivation and importance and greater sensitivity to reputational concerns. Such a pattern would be consistent with accounts that treat conventional norms as psychologically distinct from internalized moral norms (Smetana, 2013; Smetana & Yoo, 2022). If participants do not distinguish conventional norms from moral norms, this would additionally suggest there is no meaningful difference between social norms and moral norms, challenging several prominent theories of norm cognition (Bicchieri, 2006; Smetana, 2013).

Our secondary, more exploratory, research question asked whether fairness norms more closely resemble moral norms or social norms in their patterns of belief internalization. If we find that fairness norms are perceived as similarly internalized as conventional norms, this would be consistent with recent evidence suggesting that fairness is a socially conditional behavior more closely resembling social norms than moral norms. Alternatively, if fairness norms are perceived as similarly internalized as harm norms, this would provide support for their classification as moral norms.

## 2. Method

### 2.1. Participants

We tested N = 535 participants (57.2% female; M_Age_ = 41.82, SD = 13.52) from Prolific. We initially recruited 608 participants but 39 were excluded for not completing the study, and 34 were excluded for failing one of the two attention checks. Participants were compensated $3 for completing a survey that lasted approximately 17 min. This study was preregistered: https://osf.io/w2k4b/?view_only=067c5bef12144dabb4c85ee9ffb30513.

### 2.2. Design

Participants were randomly assigned to one of five behavioral domain conditions (conventional, fairness, harm, generosity, and purity) in a between-subjects design. Each participant evaluated eight behaviors belonging to their assigned domain across two parts of the study. In the first part, they completed measures of adherence importance, intrinsic motivation, and reputational concerns. In the second part, they completed additional behavioral dimension ratings, provided a definition of their assigned behavioral domain, and answered several demographic questions.

### 2.3. Procedure

Participants first provided informed consent and were introduced to the study. They were then presented with eight behaviors corresponding to their assigned behavioral domain condition—four prescriptive and four proscriptive—displayed in randomized order (see *Stimuli* in Appendix A). Participants then rated each of the behaviors on a number of measures designed to capture differences in the degree of internalization between the five behavioral domains. In the first part of the study, participants answered three sets of questions for each of the eight behaviors: two items assessing their intrinsic motivation to engage in the behavior, two items assessing whether they would be more or less likely to engage in the behavior when reputational concerns were salient, and two items assessing adherence importance^3^ (see Table 1 for the full measure text). Items within each measure were aggregated to form three primary outcome variables for analysis (see Section 3.1. *Analytic approach* below).

In the second part of the study, participants evaluated the same eight behaviors on additional dimensions, including emotional valence, injunctive normativity, descriptive normativity, morality, and expectations of punishment or reward. Participants also indicated the extent to which each behavior pertained to each of the five behavioral domains (conventional, fairness, harm, generosity, and purity; e.g., “to what extent does this behavior relate to fairness or unfairness”). Lastly, participants provided a brief definition of their assigned behavioral domain (e.g., “briefly define the concept of harm”) and completed demographic questions (age, gender, education, income, and political affiliation). All measures were administered on 100-point sliding scales unless otherwise noted. All of our analysis code, data, preregistrations, and Supplemental Material, including our study materials and all preregistered analyses, are available on the Open Science Framework: https://osf.io/ktusb/overview?view_only=9fe6f9d8ced841e0abf8252c77ce6484.

## 3. Results

### 3.1. Analytic Approach

We conducted a series of preregistered linear mixed-effects regression models predicting our key dependent variables as a function of behavioral domain (conventional, fairness, harm, generosity, and purity), using the *lme4* package in R version ‘1.1.37’ (Bates et al., 2015; R Core Team, 2024). For these analyses, we aggregated items to form three primary indices: intrinsic motivation to engage in the behaviors, perceived importance of adherence to (or avoidance of) the behaviors, and sensitivity to reputational concerns in behavioral intentions (see Table 1 for item wording). As preregistered, reputational sensitivity items were initially designed to capture two components: intentions when behavior was publicly observable (*reputation–public*) and intentions in the presence of descriptive norms (*reputation–common*).

The intrinsic motivation index (two items; α = 0.88) and the adherence importance index (two items; α = 0.92) showed high internal consistency. While participants initially answered four reputational sensitivity items per behavior (two items per subcomponent), including the preregistered reverse-coded items resulted in poor reliability, as indicated by a negative Cronbach’s alpha. Further inspection revealed that one reverse-coded item in the reputation–common subscale could not be meaningfully combined without reverse-coding and was thus conceptually misaligned with the other item.^4^ Consequently, deviating from our preregistration, we operationalized reputational sensitivity using only the two non-reverse-coded items, including the 1st (reputation–public) and 3rd (reputation–common) reputation items here, which together showed acceptable reliability (α = 0.87). For transparency, analyses using the full planned item set, including reverse-coded items, are reported in the Supplementary Materials.

We conducted 11 sets of preregistered multilevel regression models. As a manipulation check, models 1a–1e predicted ratings of each behavior dimension as a function of behavioral domain to confirm that behaviors were perceived as corresponding to their intended categories (e.g., fairness behaviors rated as most fair). We report these analyses in the supplement but note here that the behaviors largely corresponded to their hypothesized domains despite some conceptual overlap for certain behaviors—i.e., proscriptive harms were perceived as similarly harmful as proscriptive purity behaviors (see Primary Models in the Main Text and Table S3 in the Supplement for estimates for all models). Models 2a and 2b examined perceived importance by predicting adherence to prescriptive behaviors (Model 2a) and avoidance of proscriptive behaviors (Model 2b) as a function of behavioral domain. To test for differences in intrinsic motivation to engage in the behaviors across domains, Model 3 predicted intrinsic motivation by behavioral domain. Finally, Models 4a–4c examined whether sensitivity to reputational concerns varied by behavioral domain. Model 4a tested overall reputational sensitivity, Model 4b focused on behavioral intentions when actions were publicly observable, and Model 4c examined intentions in the presence of descriptive norms. Full model specifications and regression coefficients are reported in Table S3 in the supplement.

Before conducting our preregistered models, we first explored differences between the proscriptive and prescriptive norms by conducting three exploratory multilevel regression models predicting each primary dependent measure (intrinsic motivation, adherence–avoidance importance, reputational sensitivity) as a function of the interaction between behavioral domain (harm, conventional, fairness, generosity, purity; reference group = harm) and vignette valence (prescriptive, proscriptive). We compared each of these interaction models to nested models without the interaction term using Likelihood Ratio Tests. Although our preregistration specified analyzing the data collapsed across vignette valence, these exploratory models revealed multiple statistically significant behavioral domain × valence interactions (e.g., all interaction terms for intrinsic motivation were significant). Consequently, we conducted all of our preregistered analyses separately for the prescriptive and proscriptive behaviors. This resulted in each preregistered model being estimated twice: once including only prescriptive behaviors and once including only proscriptive behaviors. Additionally, because we found several norm belief covariates attenuated the effect of behavioral domain on our dependent measures, we report a series of preregistered exploratory analyses that controlled for the effect of injunctive norm beliefs, descriptive norm beliefs, emotional valence, moral judgements, and punishment evaluations. These models were identical to what we preregistered but additionally controlled for those five covariate measures to better isolate the unique effect of behavioral domain on internalization. For transparency, we report the results from the preregistered primary models without covariates in the supplement; these analyses largely replicated the covariate-adjusted results reported here (see *Preregistered Primary Models* in the Supplement). Lastly, to directly test our hypotheses regarding the relative internalization of harm and conventional norms, we re-estimated the models using alternative reference groups. Specifically, we set harm as the reference group to test whether harm behaviors were rated highest on the dependent measures and set conventional behaviors as the reference group to test whether they were rated lowest.

We also conducted a series of exploratory analyses to examine the mechanisms underlying the observed differences across behavioral domains. Specifically, we conducted a series of multilevel mediation models examining whether harm perceptions mediated the effect of behavioral domain on intrinsic motivation, controlling for the nested nature of our data at the participant level. Intrinsic motivation was selected as the focal outcome because it most clearly varied across behavioral domains. Using the *lavaan* package version ‘0.6.21’ in R (Rosseel, 2012), we estimated three mediation models comparing harm with (a) conventional norms, (b) fairness norms, and (c) purity norms. Additionally, we conducted an exploratory sentiment analysis of participants’ open-ended definitions of each behavioral domain. For each definition, we computed continuous word-level sentiment scores using the AFINN lexicon. We first tested for overall differences in sentiment across domains using a one-way ANOVA and then estimated linear regression models predicting sentiment scores from behavioral domain, with pairwise comparisons conducted using Tukey-adjusted post hoc tests. Lastly, we conducted an exploratory structural equation modeling approach testing for measurement invariance and incorporating our primary mediation hypotheses (i.e., that harm perception mediates differences between social and moral norms in measures of internalization) into a full structural model including social and moral norms as latent factors. We report these models in the supplement but summarize their results in relation to the findings of our preregistered analyses in the discussion (see *Structural Equation Models* in the Supplement for analysis approach and results).

### 3.2. Exploratory Valence Interaction Models

**Figure 1:**
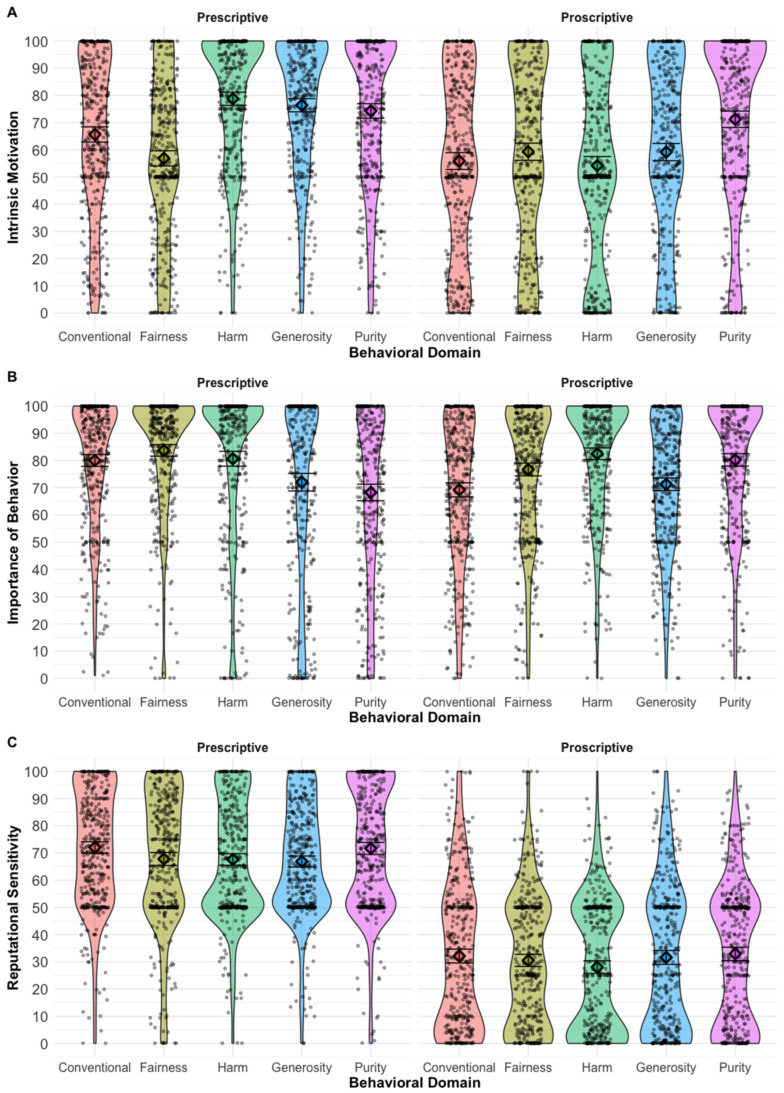
Violin plots showing the means (represented by diamonds) and distributions for the three main dependent measures by behavioral domain and vignette valence: (**A**) intrinsic motivation, (**B**) adherence importance (or avoidance, for proscriptive behaviors), and (**C**) behavioral intentions when reputational concerns are salient. Error bars show 95% confidence intervals.

We first examined whether the effect of behavioral domain on our three main dependent variables varied by vignette valence (i.e., whether the behaviors were prescriptive or proscriptive). We found that four of the five interaction terms between behavioral domain and vignette valence were significant for the adherence–avoidance importance measure, with the interaction model explaining significantly more variance than a model without the interaction term (χ^2^(4) = 118.54, *p* < 0.001; see *Vignette Valence Models* in the Supplement for full results). Similarly, for intrinsic motivation, we found all of the behavioral domain × valence interaction terms were significant (all *p*-values < 0.006), and the model including the interaction term explained significantly more variance than the model without it (χ^2^(4) = 132.67, *p* < 0.001). In contrast, there were no significant interaction terms between behavioral domain and vignette valence for reputational sensitivity when harm was set as the reference category, and the interaction model did not explain additional variance beyond the main-effects model (χ^2^(4) = 6.05, *p* = 0.19). That the effect of behavioral domain varied substantially by vignette valence for two of our three dependent measures suggested it was more appropriate to conduct our planned models by separately examining the prescriptive and proscriptive behaviors rather than collapsing across them. See Figure 1 for violin plots of participants’ responses for the three dependent measures by behavioral domain and norm valence.

### 3.3. Importance to Adhere to Prescriptive Norms or Avoid Proscriptive Norms

We then explored whether adherence importance and avoidance ratings varied across behavioral domains for the prescriptive and proscriptive behaviors such that harm behaviors would be perceived as the most important to adhere to (for prescriptive behaviors) and avoid (for proscriptive behaviors), while the conventional behaviors would be the least important to adhere to and avoid. Starting with the prescriptive norms, we found that the harm behaviors were perceived as significantly more important *to adhere* to than the generosity (B = −7.97, SE = 2.74, *p* = 0.004) and purity behaviors (B = −10.87, SE = 2.77, *p* < 0.001)—there was no difference in adherence importance between harm and conventional (B = 1.23, SE = 2.81, *p* = 0.66) or fairness behaviors (B = 5.05, SE = 2.74, *p* = 0.07), although the latter was trending toward significance (in the opposite direction of our prediction). Prescriptive conventional behaviors were rated as more important to adhere to than generosity (B = −9.21, SE = 2.81, *p* = 0.001) and purity behaviors (B = −12.10, SE = 2.70, *p* < 0.001), but did not significantly differ from fairness (B = 3.82, SE = 2.69, *p* = 0.16) or harm (B = −1.23, SE = 2.81, *p* = 0.66). Turning to the proscriptive behaviors, we found a different pattern of results when examining which behaviors were perceived as the most important *to avoid*. The harm behaviors were rated as the most important to avoid, more than conventional (B = −10.82, SE = 2.17, *p* < 0.001), fairness (B = −5.48, SE = 2.15, *p* = 0.011), and generosity behaviors (B = −10.57, SE = 2.17, *p* < 0.001), but did not significantly differ from the purity behaviors (B = −0.18, SE = 2.16, *p* = 0.94). The proscriptive conventional behaviors were rated as less important to avoid than fairness (B = 5.33, SE = 2.13, *p* = 0.012), harm (B = 10.82, SE = 2.17, *p* < 0.001), and purity behaviors (B = 10.64, SE = 2.12, *p* < 0.001), but did not differ significantly from the generosity behaviors (B = 0.25, SE = 2.15, *p* = 0.91). See Table 2 for a visual depiction of our findings for all three dependent measures of belief internalization.

### 3.4. Intrinsic Motivation to Engage in the Behavior

We next examined whether participants were most intrinsically motivated to engage in harm behaviors and least intrinsically motivated to engage in conventional behaviors. Starting with the prescriptive norms, we found that harm behaviors were perceived as more intrinsically motivating than conventional (B = −9.50, SE = 2.78, *p* < 0.001) or fairness behaviors (B = −18.02, SE = 2.72, *p* < 0.001), but did not differ significantly from generosity (B = −2.26, SE = 2.72, *p* = 0.41) or purity (B = −1.12, SE = 2.75, *p* = 0.68). Conventional behaviors were perceived as significantly more intrinsically motivated than fairness behaviors (B = −8.52, SE = 2.67, *p* = 0.002) and significantly less intrinsically motivated than harm (B = 9.50, SE = 2.78, *p* < 0.001), generosity (B = 7.24, SE = 2.78, *p* = 0.009), and purity behaviors (B = 8.39, SE = 2.68, *p* = 0.002). We found a different pattern of results when examining proscriptive norms. Namely, proscriptive harm behaviors were perceived as significantly less intrinsically motivating than purity behaviors (B = 13.61, SE = 3.78, *p* < 0.001) but did not otherwise differ from conventional (B = −1.73, SE = 3.78, *p* = 0.65), fairness (B = 3.20, SE = 3.75, *p* = 0.39), or generosity behaviors (B = 2.41, SE = 3.79, *p* = 0.52). Proscriptive conventional behaviors were also rated as significantly less intrinsically motivated than purity behaviors (B = 15.35, SE = 3.73, *p* < 0.001) but did not differ significantly from fairness (B = 4.93, SE = 3.71, *p* = 0.18), harm (B = 1.73, SE = 3.78, *p* = 0.65), or generosity behaviors (B = 4.15, SE = 3.76, *p* = 0.27).

### 3.5. Effect of Reputational Concerns on Behavioral Intentions

When reputational concerns were salient, participants reported being less likely to engage in prescriptive harm behaviors than in conventional (B = 9.77, SE = 2.21, *p* < 0.001), fairness (B = 6.20, SE = 2.16, *p* = 0.004), and purity (B = 8.65, SE = 2.18, *p* < 0.001) behaviors, but not generosity behaviors (B = 0.03, SE = 2.16, *p* = 0.99). Participants’ intentions to engage in prescriptive conventional behaviors were more sensitive to reputational concerns than harm (B = −9.77, SE = 2.21, *p* < 0.001) and generosity (B = −9.74, SE = 2.20, *p* < 0.001), but did not differ significantly from fairness (B = −3.57, SE = 2.12, *p* = 0.09) or purity (B = −1.12, SE = 2.13, *p* = 0.60). Next, turning to the results for the proscriptive norms, we found that participants’ intentions to engage in harm behaviors when reputation was salient did not differ significantly from conventional, fairness, generosity, or purity behaviors (all *p*-values > 0.51). Likewise, participants’ intentions to engage in proscriptive conventional behaviors when reputation was salient did not differ significantly from fairness, harm, generosity, or purity (all *p*-values > 0.29).

We next examined each of the two reputational concern measures individually: reputation–public and reputation–common. When examining differences in behavioral intentions when the behavior would be public and common knowledge, we found that participants reported being less likely to engage in prescriptive harm behaviors than the conventional (B = 10.20, SE = 2.33, *p* < 0.001), fairness (B = 7.10, SE = 2.28, *p* = 0.002), and purity behaviors (B = 11.06, SE = 2.30, *p* < 0.001), but not generosity behaviors (B = 0.03, SE = 2.28, *p* = 0.99). Participants reported they were more likely to engage in prescriptive conventional behaviors when their behavior would be public and common knowledge as compared to harm (B = −10.20, SE = 2.23, *p* < 0.001) and generosity (B = −10.17, SE = 2.33, *p* < 0.001) behaviors—the difference was trending toward significance for fairness (B = −3.09, SE = 2.24, *p* = 0.17), and did not significantly differ for purity behaviors (B = 0.86, SE = 2.25, *p* = 0.70). When examining the proscriptive behaviors, we found that participants were not more likely to report engaging in harm behaviors when they were common knowledge than conventional, fairness, generosity, or purity behaviors (all *p*-values > 0.17). Likewise, there were no differences in intentions between proscriptive conventional behaviors and fairness, harm, generosity, or purity (all *p*-values > 0.17).

Turning to differences in behavioral intentions when the behavior was a descriptive norm, we found that participants’ intentions were significantly lower for prescriptive harm behaviors than conventional (B = 9.39, SE = 2.34, *p* < 0.001), fairness (B = 5.38, SE = 2.29, *p* = 0.019), and purity behaviors (B = 6.33, SE = 2.30, *p* = 0.006)—there was no difference relative to generosity behaviors (B = 0.008, SE = 2.28, *p* = 0.99). Behavioral intentions for prescriptive conventional behaviors were more sensitive to descriptive norms than for harm (B = −9.39, SE = 2.34, *p* < 0.001) and generosity behaviors (B = −9.38, SE = 2.34, *p* < 0.001); the difference with fairness was trending toward significance (B = −4.01, SE = 2.24, *p* = 0.07), and there was no difference compared to purity (B = −3.06, SE = 2.25, *p* = 0.17). When looking at proscriptive behaviors, we found no differences in behavioral intention sensitivity to descriptive norms although proscriptive harms were rated as marginally less sensitive than fairness behaviors (B = 5.05, SE = 2.82, *p* = 0.07). There were no significant differences between the proscriptive harm and conventional, generosity, or purity behaviors (all *p*-values > 0.64). Nor were there differences in behavioral intention sensitivity to descriptive norms for proscriptive conventional behaviors as compared to fairness, harm, generosity, or purity behaviors (all *p*-values > 0.18).

### 3.6. Mechanisms Underlying Differences Between Behavioral Domains

We next conducted a series of exploratory analyses to investigate the potential mechanisms underlying differences across behavioral domains. Namely, the harm perception mediation models explored whether differences in perceptions of harm explained differences in intrinsic motivation, the outcome measure that conceptually corresponds most closely to internalization. In the first mediation model, we tested whether harm perceptions mediated differences in intrinsic motivation between harm and conventional behaviors. The indirect effect was significant (b = 7.01, SE = 1.25, *p* < 0.001), explaining 10.9% of the variance in intrinsic motivation between harm and conventional behaviors and indicating that differences in perceptions of harm partially explained the observed differences in intrinsic motivation between the two behavioral domains. We then tested whether harm perceptions mediated differences in intrinsic motivation between the harm and fairness behaviors. The indirect effect was again significant (b = 5.55, SE = 1.13, *p* < 0.001), explaining 8.5% of the total difference in intrinsic motivation, suggesting that harm perceptions partially accounted for the reported motivational gap between harm and fairness norms. Lastly, we examined whether harm perception mediated differences between harm and purity behaviors. In this case, the indirect effect of harm perceptions on intrinsic motivation was not statistically significant, though it trended toward significance (b = 1.21, SE = 0.71, *p* = 0.086), accounting for 1.9% of the difference in intrinsic motivation. This pattern suggests that differences in perceived harm played a limited role in explaining motivational differences between harm and purity behaviors.

Lastly, we investigated the affective sentiment (i.e., positive or negative) expressed in participants’ definitions of each behavioral domain. Results of the one-way ANOVA revealed a significant effect of behavioral domain on sentiment (*F*(4, 350) = 166.5, *p* < 0.001). Harm behaviors were described with markedly negative sentiment (M = −2.14, SD = 0.47), significantly lower than all other categories (*p*-values < 0.001). In contrast, generosity behaviors elicited the most positive sentiment (M = 1.66, SD = 1.09), followed closely by conventional (M = 1.13, SD = 0.92), purity (M = 1.02, SD = 1.52) and fairness (M = 1.11, SD = 1.28). Moreover, generosity definitions were significantly more positive than those for fairness (*p* = 0.047), purity (*p* = 0.006), and to a marginal extent, conventional (*p* = 0.07) behaviors. The remaining domains did not differ significantly from one another (*p*-values > 0.98).

## 4. Discussion

The present research examined how people perceive different types of norms commonly described as social or moral, with the goal of clarifying whether moral norms are perceived as psychologically distinct from social norms and, if so, what distinguishes them. Across five normative domains—conventional norms, fairness, harm, generosity, and purity—we found systematic differences in how behaviors were perceived along dimensions theorized to reflect norm internalization, including intrinsic motivation, perceived importance of adherence or avoidance, and sensitivity to reputational concerns. Overall, we found significant differences across behavioral domains on several measures, suggesting that harm norms may be more internalized than other moral domains like fairness. However, these differences did not always consistently align with our predictions and varied substantially depending on the valence of the behaviors. For example, when examining intrinsic motivation to engage in the behaviors, we found that prescriptive harm behaviors were perceived as more intrinsically motivating than fairness and conventional behaviors, but not purity or generosity behaviors. In contrast, proscriptive harm behaviors were perceived as less intrinsically motivating than purity behaviors, but not other behavioral domains. We discuss these findings for each of our research questions below.

### 4.1. Are Moral Norms Distinct from Social Norms?

A central unresolved question in the study of norm cognition is whether social and moral norms constitute psychologically distinct categories of social information or whether they reflect different labels applied to a common underlying phenomenon. While some previous work has conflated moral and social norms (Lindström et al., 2018; Mulder, 2018; Smith & Masser, 2012), several theoretical accounts of norms such as Social Domain Theory (SDT) and Bicchieri’s social conditionality theory (SCT) propose there is a categorical distinction between social and moral norms (Bicchieri, 2006; Smetana & Yoo, 2022). The present findings provide only partial support for these latter theories. Across multiple indicators theorized to reflect norm internalization, we observed systematic differences among behavioral domains, suggesting that there are important differences in how people perceive various types of social and moral norms. However, these differences did not consistently align with a sharp moral–social divide.

We consolidate our results into three main findings. First, harm norms were generally perceived as the most internalized moral norms, suggesting that harm is the most representative category of moral norms. Specifically, prescriptive harm behaviors were descriptively rated as the most intrinsically motivating (significantly more so than conventional and fairness behaviors) and the least sensitive to reputational concerns (significantly less so than all domains other than generosity). Additionally, proscriptive harm behaviors were rated as significantly more important to avoid than all behavioral domains other than purity. These findings align with prior work conceptualizing harm as a prototypical moral norm underlying a broad range of moral judgments (Schein & Gray, 2018; Sousa et al., 2009). However, at the same time, harm norms frequently exhibited a complex and varied pattern of internalization such that they were largely context-dependent, differing between prescriptive and proscriptive contexts and relative to other moral domains.

The second main finding was that conventional norms were perceived as relatively less internalized (e.g., less intrinsically motivating, more sensitive to reputational concerns) and thus more socially conditional than most of the moral behaviors studied (with the notable exception of fairness). Namely, prescriptive conventional behaviors were rated as less intrinsically motivating than all moral norms other than fairness, behavioral intentions for prescriptive conventional norms were more sensitive to reputational concerns than those for harm and generosity behaviors, and proscriptive conventional behaviors were rated as less important to avoid than all moral domains other than generosity. This suggests that conventional norms are best conceptualized as social norms and differ in several key respects from the different domains of moral norms studied here. However, with that said, conventional norms did not consistently differ from every type of moral norm—while we found substantial differences between conventional and harm behaviors across our measures, these effects were smaller or inconsistent for other types of moral behaviors like generosity. This suggests there is not a clear boundary between social and moral norms for many commonly studied moral behaviors like generosity and fairness.

The evidence for reputational sensitivity indicates high domain-specific variation but without a clear moral–social divide. While prescriptive harm norms appeared less influenced by reputational concerns and conventional norms somewhat more so, this pattern did not extend to proscriptive contexts. Taken together, these findings offer only partial support for the claim that moral norms are less reputation-dependent than social norms and suggest that sensitivity to reputational concerns may be the least associated with belief internalization out of our three measures. More generally, our results are consistent with theoretical accounts emphasizing the role of reputation and impression management in social cognition (Barclay, 2012; Goffman, 1959/1990). However, given the measurement limitations related to the assessment of reputational concerns, we cannot make any strong conclusions here and thus it remains unclear whether certain domains are reliably more reputation-sensitive than others.

Our third main finding concerned the results of our exploratory mediation analyses. These findings highlight the important role perceptions of harm play in underlying differences in internalization between social norms and various moral norm domains. Namely, we found that harm perceptions partially mediated the differences in intrinsic motivation between conventional and harm behaviors and between the fairness and harm behaviors. These mediation results suggest that harm behaviors were perceived as more intrinsically motivating to engage in than conventional or fairness behaviors in part because they were perceived as more harmful. This suggests that at least some of the differences we observed between social conventional and moral norm domains can be attributed to differences in perceptions of harm. These findings support the claim that harm is one of the key dimensions underlying moral judgements of normative behavior. Specifically, our harm perception findings align with Gray’s Dyadic theory of morality, which posits that harm is the central domain underlying moral judgements (contrasting with pluralist accounts such as MFT which posit multiple discrete moral domains). Moreover, our results suggest that the extent to which participants internalized the other moral behaviors may depend on perceptions of harm—e.g., the greater the harm perceptions, the more internalized the behavior is.

Why might internalization differ between social and moral norm domains and along perceptions of harm? One possibility is that we may be innately predisposed to acquire moral beliefs for harm behaviors because evolutionarily, harm-related concerns would have had a more direct impact on fitness than other moral domains such as fairness or generosity (Cosmides & Tooby, 1994; Sheskin & Santos, 2012). Alternatively, harm may be relatively more internalized if children receive more experience and social information related to harmful behaviors than other moral norms. However, that children receive more information about and experience with harm-related norms may itself reflect its innate roots in moral cognition. Answering this question is beyond the scope of the present work but highlights the necessity for additional research on domain differences in the ontology of belief internalization.

Additionally, to address concerns about the construct validity of the internalization measures and test whether social and moral norms represent psychometrically distinct dimensions, we conducted exploratory measurement invariance tests and a full structural mediation model using structural equation modeling. A two-factor model (intrinsic motivation; norm adherence importance) fit the data well and demonstrated metric invariance across social and moral norm types (ΔCFI = −0.001, ΔRMSEA = −0.001), confirming that the constructs are measured equivalently across norm domains. The full SEM model with harm as a mediator found converging support for one of the paper’s main findings—that harm perception is a key mechanism underlying differences in norm internalization across behavioral domains—while also adjusting for the asymmetric role of harm perception across the two included dimensions of internalization (i.e., intrinsic motivation and adherence–avoidance importance). Moreover, we found evidence of full mediation: the direct effect of social–moral norms on internalization was not significant when including the indirect effect of harm perceptions (e.g., social–moral → harm perceptions → importance and intrinsic motivation). This suggests that participants’ greater intrinsic motivation to comply with the moral norm behaviors was entirely explained by their perception of those behaviors as more harmful. This finding directly supports the role of harm perception as a psychological mechanism linking moral norms to belief internalization and is consistent with harm-based accounts of moral judgment (Cushman, 2013; Gray et al., 2012). Full details of all models are reported in the Supplementary Materials.

In sum, our results indicate that while people perceive social norms differently from moral norms, only harm norms were perceived to consistently differ from social norms (i.e., conventional norms), suggesting harm is the most internalized, and thus most representative, moral norm. Importantly, however, our finding that norm internalization varied continuously across domains and normative contexts suggests that differences between moral and social norms are better understood as graded differences in degree rather than categorical differences in kind. More generally, our findings inform previous theories of moral cognition that posit discrete moral domains (such as MFT; Graham et al., 2011). Namely, with the exception of harm, we failed to find clear differences in internalization across moral domains suggesting that, while there may be domain differences in moral cognition, they may be undergirded by the same cognitive mechanisms.

### 4.2. Is Fairness a Moral Norm?

Another question we explored in the present work was whether fairness is a social or moral norm. A large literature has investigated the relationship between fairness and morality, with some academics, such as proponents of MFT and MAC, arguing that fairness is a distinct domain of morality (Curry, 2016; Graham et al., 2011). While fairness is undoubtedly central to human morality, and many psychologists assume fairness norms fall in the moral domain (Baumard, 2016; Lindström et al., 2018; Smetana & Ball, 2018; Turiel et al., 1987), recent research casts doubt on the validity of categorizing fairness as a moral norm comparable to harm or purity. Namely, this work finds that people view fairness differently from the more prototypical moral norm of harm—for example, both children and adults perceive fairness norm violations as less severe and deserving of punishment than harm norm violations (Yucel & Vaish, 2025; Yucel et al., 2022)—while other work suggests people view fairness norms more similarly to conventional norms than moral norms like harm (Deutchman et al., 2024; Yucel & Vaish, 2025). Our findings contribute to this debate by showing that fairness norms are not perceived in the same way as harm norms across multiple indicators of belief internalization. For example, prescriptive fairness behaviors were perceived as less intrinsically motivating than conventional and harm behaviors. Similarly, proscriptive harm behaviors—akin to harm norm violations—were perceived as more important to avoid than violations of both fairness and conventional norms. Participants also reported greater sensitivity to reputational concerns in their intentions to engage in prescriptive fairness than harm behaviors, indicating that fairness norms may be more influenced by social information. These results align with recent evidence which finds that behavioral intentions and injunctive norm beliefs for fairness behaviors are more sensitive to descriptive norm information than those for harm (Deutchman et al., 2024).

Moreover, we also found that the prescriptive fairness behaviors were simultaneously rated as descriptively highest in importance to adhere to and the least intrinsically motivated (i.e., the most extrinsically motivated) of the behavioral domains. That these fairness behaviors were perceived as important, yet also more extrinsically motivated, provides additional evidence that fairness norms are socially conditional on others’ behavior (and thus differ from moral norms like harm). Consequently, this suggests that fairness may correspond more closely with Bicchieri’s conceptualization of social norms—where conformity is conditional on what others do and expect—than moral norms, which should be insensitive to beliefs about others’ behavior or expectations (Bicchieri, 2006, 2016). Why are fairness norms relatively less internalized, and thus more socially conditional, than harm norms? As mentioned above, one possibility is that humans are predisposed to more readily acquire certain moral norms of harm (e.g., unprovoked harm is wrong) as compared to other moral domains like fairness because they have historically conveyed a more direct fitness advantage. Thus, while we may be predisposed to broadly acquire fairness norms, there may be relatively more cognitive flexibility in which fairness-related norms are acquired—as evidenced by greater cross-cultural variability in fairness than harm norms (Barrett et al., 2016; Yau & Smetana, 2003). However, with that said, our current findings are unable to directly address this possibility.

In sum, our results add to recent work examining the differences between fairness and other moral norms which suggests there are important differences in moral cognition for fairness as compared to other moral domains like harm. More broadly, our findings suggest that fairness may be better conceptualized as a social norm than a moral norm.

### 4.3. Limitations and Future Directions

The present work has several limitations. First, because internalization is, by definition, an internal psychological process that cannot be directly observed (Gavrilets & Richerson, 2017; Gintis, 2003), our self-report measures were imperfect proxies for capturing differences in internalization across the various types of social and moral norms studied here. Future work should investigate the role of internalization in moral norms using more implicit measurement approaches, such as reaction time measures. Second, although we had planned to reverse-code two of the four reputation items, the Cronbach’s alphas for both reputation indices were negative, suggesting that it was not statistically appropriate to reverse score the items and combine them into an index. In light of this, we reported the results for the reputation measures excluding the reverse-coded items in the main text and reported the results for our preregistered models including the planned reverse-coded items in the supplement. Consequently, while we observed differences between the behavioral domains in sensitivity to reputational concerns, the conclusions we can draw are hampered by the methodological limitations of our measurement approach. Future work should use improved self-report measures and assess real norm compliance behavior to better understand whether people are differentially influenced by the presence of common knowledge and descriptive norms for different types of social and moral norms. Third, we had not initially anticipated finding such distinct patterns of results across vignette valence (i.e., prescriptive or proscriptive) and as a result, had to deviate from our planned analysis approach to jointly examine the effect of behavioral domain on our dependent measures. That we found substantially different patterns of results depending on the emotional valence of the norm highlights the importance of distinguishing prescriptive (that we should engage in) from proscriptive behaviors (that we should avoid) in social norm research. Future work should be mindful of these differences when investigating norm cognition.

A fourth limitation concerns our sample, namely that our data were collected using an online convenience sample. While previous research has found few differences in studies conducted in lab or online (Amir et al., 2012; Horton et al., 2011), the generalizability of our findings remains uncertain. Specifically, because participants were exclusively recruited from the United States—and thus constitute a WEIRD sample (Henrich et al., 2010)—the extent to which these findings apply to other cultural contexts is unclear. Future research should examine the robustness of these findings across diverse populations to determine whether patterns of norm internalization systematically vary across societies. Fifth, while we did not assess the role of affect in norm cognition, past work suggests that affect plays an important proximate role in underlying normative behavior (Packard & Schultz, 2023)—future work should directly explore the relationship between affect and norm cognition, and specifically whether social and moral norms are associated with distinct emotions and affective states. Finally, despite controlling for possible confounds by controlling for norm-related covariates (e.g., injunctive norm beliefs), it is possible that our results may hinge on the specific behaviors included as stimuli and thus might not fully generalize to different behaviors.

### 4.4. Conclusions

Together, our findings advance our understanding of norms by investigating a key psychological process underlying moral norm cognition and distinguishing moral norms from social norms. Namely, our results suggest that the extent to which beliefs are internalized varies depending on the moral domain of the behavior but offer inconclusive evidence for differences in internalization between specific moral norm domains. Overall, our results suggest that the harm behaviors are the most internalized of the moral norms studied here and are particularly distinct from conventional norms. Our findings also inform our understanding of the relationship between fairness and moral norms, providing additional evidence that fairness norms may be perceived more similarly to socially conditional social norms (e.g., conventional norms) than socially independent moral norms like harm. In sum, our results both inform our understanding of the conceptual distinction between social and moral norms and raise new questions about the role of internalization in underlying moral and normative behavior.

## Figures and Tables

**Table 1:** Table with measure text. All items were on a 100-pt sliding scale. See Appendix A for anchors and scale points.

Measure	Measure Text
Intrinsic motivation	To what extent would you engage in the behavior because others expect you to or because it’s your personal choice? To what extent would you engage in the behavior because that’s what you’re supposed to do or because you actually want to?
Reputational concern–public	Would you be more or less likely to engage in the behavior if you were around other people at the time or if your actions would be publicly known?
Reputational concern–common	1. How likely would you be to engage in the behavior if most people you know are engaging in it?
Adherence importance	How important is it to you to engage in this behavior? How strongly do you believe that people should engage in this behavior?

**Table 2:** Table displaying how the three dependent measures of internalization differed for the conventional and harm domains compared to all five moral domains. ✓ indicates a statistically significant difference in the predicted direction (*p* < 0.05), ❖ indicates the effect was statistically significant in the opposite pattern than predicted (*p* < 0.05), *ns* indicates the effect was not significant in the predicted direction (*p* > 0.05).

Domain	Conventional	Fairness	Generosity	Harm	Purity
DV 1-Norm importance (prescriptive: adherence; proscriptive: avoidance)					
Prescriptive					
Conventional	-	*ns*	❖	*ns*	❖
Harm	*ns*	*ns*	✓	-	✓
Proscriptive					
Conventional	-	✓	*ns*	✓	✓
Harm	✓	✓	✓	-	*ns*
DV 2-Intrinsic motivation					
Prescriptive					
Conventional	-	❖	✓	✓	✓
Harm	✓	✓	*ns*	-	*ns*
Proscriptive					
Conventional	-	*ns*	*ns*	*ns*	✓
Harm	*ns*	*ns*	*ns*	-	❖
DV 3-Reputational sensitivity of behavioral intentions					
Prescriptive					
Conventional	-	*ns*	✓	✓	*ns*
Harm	✓	✓	*ns*	-	✓
Proscriptive					
Conventional	-	*ns*	*ns*	*ns*	*ns*
Harm	*ns*	*ns*	*ns*	-	*ns*

## Data Availability

All of our code, materials, and data are publicly available online at the Open Science Framework: https://osf.io/ktusb/overview?view_only=194d57b5a3ee4fa4984264508c68259e.

## Notes

1 With that said, social conventions in SDT closely correspond to many types of social norms frequently studied in the literature (e.g., waiting in a queue, community dress codes, etc.; Fagundes, 2017; Malovicki-Yaffe et al., 2023).

2 To be clear, our focus is specifically on the psychological processes and cognitive mechanisms that may underlie and differentiate social and moral norms, rather than on their ontological status.

3 Participants were asked about the importance of adhering to prescriptive behaviors and the importance of *avoiding* proscriptive behaviors.

4 I.e., it does not make sense conceptually to combine an item measuring behavioral intentions to engage in the behavior when common (“how likely would you be to engage in the behavior if most people you know are engaging in it”) with an item measuring intentions when the behavior is uncommon (“how likely would you be to engage in the behavior if most people you know are not engaging in it”) for the same behavior without reverse scoring the latter.

## Supplementary Materials

The following supporting information can be downloaded at: https://www.mdpi.com/article/10.3390/bs16050774/s1, Table S1. Table showing averages and standard deviations for the five dimension ratings for each behavioral domain and the Pearson’s correlation coefficient across dimension ratings. Table S2. Table showing averages and standard deviations for the five dimension ratings for each behavioral domain separated by norm valence. Table S3. Model parameters and estimates. Models 1a-4c were our primary analyses. All models included the random effects of participant and behavior ID and controlled for covariates (emotional valence, descriptive norms, injunctive norms, punishment-reward evaluations, and moral judgements). For models 1a-e we set the reference group corresponding to the relevant dimension comparisons (e.g., fairness was the reference group when comparing the extent to which the behaviors pertained to fairness), while for models 2-4c, harm was set as the reference group. Table S4. Model table with path estimates for the harm perception mediation model comparing harm with conventional behaviors. Table S5. Model table with path estimates for the harm perception mediation model comparing harm with fairness behaviors. Table S6. Model table with path estimates for the harm perception mediation model comparing harm with purity behaviors. Table S7. Model table with fit indices for the three measurement invariance models. Table S8. Model table of estimates for the full structural equation model examining the indirect effect of norm type on internalization through harm perceptions. Figure S1. Violin plots showing the means and distributions for the five behavioral dimension measures (social conventions, fairness, generosity, harm, and purity) by behavioral domain and norm valence. Figure S2. Violin plot showing net sentiment scores for each behavioral domain, collapsing across prescriptive and proscriptive norms. Figure S3. Path diagram model of the full structural equation model with harm perception as a mediator. (Breusch & Pagan, 1980; Chen, 2007; Cheung & Rensvold, 2002; Little et al., 1999; MacKinnon et al., 2007; Satorra & Bentler, 2001) are cited in Supplementary Materials.

## Appendix A. Study Materials

Measures

All measures were on 100-point sliding scales.

*1. Intrinsic Motivation*

(1) To what extent would you engage in the behavior because others’ expect you to or because it’s your personal choice? (0—Because others’ expect me to, 100—Because it’s my personal choice)
(2) To what extent would you engage in the behavior because that’s what you’re supposed to do or because you actually want to? (0—Because you’re supposed to, 100—Because I want to)

*2. Adherence–Avoidance Importance*

*Proscriptive—Avoidance*

(1) How important is it to you to avoid engaging in the behavior? (0—Not at all important, 100—Extremely important)
(2) How strongly do you believe that people should avoid engaging in this behavior? (0—Not strongly at all, 100—Extremely strongly)

*Prescriptive—Adherence*

(1) How important is it to you to engage in the behavior? (0—Not at all important, 100—Extremely important)
(2) How strongly do you believe that people should engage in this behavior? (0—Not strongly at all, 100—Extremely strongly)

*3. Reputational Sensitivity*

*Reputation–Public*

(1) Would you be more or less likely to engage in the behavior if you were around other people at the time or if your actions would be publicly known? (0—Less likely, 50—Neither less nor more likely, 100—More likely)
(2) If you could engage in the behavior without anyone knowing or ever finding out, would you be more or less likely to do it? (0—Less likely, 50—Neither less nor more likely, 100—More likely)

*Reputation–Common*

(3) How likely would you be to engage in the behavior if most people you know are engaging in it? (0—Less likely, 50—Neither less nor more likely, 100—More likely)
(4) How likely would you be to engage in the behavior if most people you know are not engaging in it? (0—Less likely, 50—Neither less nor more likely, 100—More likely)

*4. Norm Covariates*

(1) *Valence*: In your opinion, how emotionally positive or negative is this behavior? (-50—Extremely negative, 0—Neither negative nor positive, 50—Extremely positive)
(2) *Injunctive Norm Beliefs*: In your opinion, how much do other people approve or disapprove of this behavior when they are in the relevant situation? (-50—Entirely disapprove, 0—Neither disapprove nor approve, 50—Entirely approve)
(3) *Descriptive Norm Beliefs*: In your opinion, how many people in your community do this behavior when they are in the relevant situation? (-50—No one, 0—Some people, 50—Everyone)
(4) *Moral Judgements*: In your opinion, how moral or immoral is it to do this behavior? (-50—Extremely immoral, 0—Neither immoral nor moral, 50—Extremely moral)
(5) *Punishment–Reward*: In your opinion, would the person who does this behavior get rewarded or punished by others? (-50—Definitely punished, 0—Neither punished nor rewarded, 50—Definitely rewarded)

*Behavior Dimension Ratings*

Participants were asked to “rate the extent to which the behavior relates to the following categories: social conventions, fairness (or unfairness), harm (or care), generosity (or selfishness), and purity (or impurity). Note that some of the categories will not be relevant for all behaviors.” (0—Not at all relevant, 100—Completely relevant)

(1) Social Convention: To what extent does the behavior relate to social conventions?
(2) Fairness: To what extent does the behavior relate to fairness or unfairness?
(3) Harm: To what extent does the behavior relate to harm or care?
(4) Purity: To what extent does the behavior relate to purity or impurity?
(5) Generosity: To what extent does the behavior relate to generosity or selfishness

Stimuli

Conventional:

Prescriptive

1. Wearing fashionable clothes
2. Shaking hands when meeting a new person
3. Being on time to meet up with friends
4. Making eye contact with the person you’re talking to

Proscriptive

1. Dressing inappropriately for a formal occasion
2. Speaking loudly in a library
3. Listening to music without headphones on public transit
4. Driving over the speed limit

Fairness:

Prescriptive

1. Queueing at the checkout when shopping
2. Splitting the bill based on what each person ordered
3. Equally contributing to a group project
4. Remaining neutral in a disagreement between friends

Proscriptive

1. Cutting a line at checkout
2. Leaving dirty dishes in a shared kitchen
3. Cheating on a test or exam
4. Driving on the shoulder of the highway to get around traffic

Harm:

Prescriptive

1. Rescuing an injured animal
2. Buying a homeless person food
3. Consoling a grieving friend
4. Taking care of a friend or family member with a serious illness

Proscriptive

1. Starting a false rumor about someone
2. Spanking your child
3. Intentionally tripping someone
4. Verbally harassing a store employee

Generosity:

Prescriptive

1. Giving money to charity
2. Donating blood
3. Volunteering at a food bank
4. Contributing to a GoFundMe to cover someone’s medical bills

Proscriptive

1. Parking in a handicap spot without a permit
2. Taking office supplies home from work for your personal use
3. Stealing a coworker’s idea and presenting it as your own
4. Stockpiling essential items during an emergency

Purity:

Prescriptive

1. Washing your hands after using the bathroom
2. Maintaining personal hygiene
3. Choosing natural and organic products
4. Staying home when sick

Proscriptive

1. Engaging in promiscuous sexual behavior
2. Consuming excessive amounts of junk food
3. Eating food that fell on the ground
4. Doing illicit drugs

## References

[B1-behavsci-16-00774] Amir O., Rand D. G., Gal Y. K. (2012). Economic games on the internet: The effect of $1 stakes. PLoS ONE.

[B2-behavsci-16-00774] Atari M., Haidt J., Graham J., Koleva S., Stevens S. T., Dehghani M. (2023). Morality beyond the WEIRD: How the nomological network of morality varies across cultures. Journal of Personality and Social Psychology.

[B3-behavsci-16-00774] Barclay P. (2012). Harnessing the power of reputation: Strengths and limits for promoting cooperative behaviors. Evolutionary Psychology.

[B4-behavsci-16-00774] Barrett H. C., Bolyanatz A., Crittenden A. N., Fessler D. M., Fitzpatrick S., Gurven M., Henrich J., Kanovsky M., Kushnick G., Pisor A., Scelza B. A., Stich S., von Rueden C., Zhao W., Laurence S. (2016). Small-scale societies exhibit fundamental variation in the role of intentions in moral judgment. Proceedings of the National Academy of Sciences of the United States of America.

[B5-behavsci-16-00774] Bates D., Mächler M., Bolker B., Walker S. (2015). Fitting linear mixed-effects models using lme4. Journal of Statistical Software.

[B6-behavsci-16-00774] Baumard N. (2016). The origins of fairness: How evolution explains our moral nature.

[B7-behavsci-16-00774] Baumard N., André J. B., Sperber D. (2013). A mutualistic approach to morality: The evolution of fairness by partner choice. Behavioral and Brain Sciences.

[B8-behavsci-16-00774] Berneri C., Larcom S., Peng C., She P. W. (2024). The impact of law on moral and social norms: Evidence from facemask fines in the UK. European Journal of Law and Economics.

[B9-behavsci-16-00774] Bicchieri C. (2006). The grammar of society: The nature and dynamics of social norms.

[B10-behavsci-16-00774] Bicchieri C. (2016). Norms in the wild: How to diagnose, measure, and change social norms.

[B11-behavsci-16-00774] Blake P. R., McAuliffe K., Corbit J., Callaghan T. C., Barry O., Bowie A., Kleutsch L., Kramer K. L., Ross E., Vongsachang H., Wrangham R., Warneken F. (2015). The ontogeny of fairness in seven societies. Nature.

[B12-behavsci-16-00774] Breusch T. S., Pagan A. R. (1980). The Lagrange multiplier test and its applications to model specification in econometrics. The Review of Economic Studies.

[B13-behavsci-16-00774] Chakroff A., Dungan J., Koster-Hale J., Brown A., Saxe R., Young L. (2016). When minds matter for moral judgment: Intent information is neurally encoded for harmful but not impure acts. Social Cognitive and Affective Neuroscience.

[B14-behavsci-16-00774] Chen F. F. (2007). Sensitivity of goodness of fit indexes to lack of measurement invariance. Structural Equation Modeling: A Multidisciplinary Journal.

[B15-behavsci-16-00774] Cheung G. W., Rensvold R. B. (2002). Evaluating goodness-of-fit indexes for testing measurement invariance. Structural Equation Modeling.

[B16-behavsci-16-00774] Cosmides L., Tooby J. (1994). Origins of domain specificity: The evolution of functional organization. Mapping the mind: Domain specificity in cognition and culture.

[B17-behavsci-16-00774] Curry O. S. (2016). Morality as cooperation: A problem-centred approach. The evolution of morality.

[B18-behavsci-16-00774] Curry O. S., Chesters M. J., Van Lissa C. J. (2019). Mapping morality with a compass: Testing the theory of ‘morality-as-cooperation’ with a new questionnaire. Journal of Research in Personality.

[B19-behavsci-16-00774] Cushman F. (2013). Action, outcome, and value: A dual-system framework for morality. Personality and Social Psychology Review.

[B20-behavsci-16-00774] Deutchman P., Kraft-Todd G., Young L., McAuliffe K. (2024). People update their injunctive norm and moral beliefs after receiving descriptive norm information. Journal of Personality and Social Psychology.

[B21-behavsci-16-00774] Dungan J. A., Chakroff A., Young L. (2017). The relevance of moral norms in distinct relational contexts: Purity versus harm norms regulate self-directed actions. PLoS ONE.

[B22-behavsci-16-00774] Dungan J. A., Young L. (2019). Asking ‘why?’ enhances theory of mind when evaluating harm but not purity violations. Social Cognitive and Affective Neuroscience.

[B23-behavsci-16-00774] Elster J. (2006). Fairness and norms. Social Research: An International Quarterly.

[B24-behavsci-16-00774] Essler S., Christner N., Becher T., Paulus M. (2023). The ontogenetic emergence of normativity: How action imitation relates to infants’ norm enforcement. Journal of Experimental Child Psychology.

[B25-behavsci-16-00774] Fagundes D. (2017). The social norms of waiting in line. Law & Social Inquiry.

[B26-behavsci-16-00774] Fehr E., Fischbacher U. (2004). Social norms and human cooperation. Trends in Cognitive Sciences.

[B27-behavsci-16-00774] FeldmanHall O., Son J. Y., Heffner J. (2018). Norms and the flexibility of moral action. Personality Neuroscience.

[B28-behavsci-16-00774] Gavrilets S., Richerson P. J. (2017). Collective action and the evolution of social norm internalization. Proceedings of the National Academy of Sciences of the United States of America.

[B29-behavsci-16-00774] Gintis H. (2003). The hitchhiker’s guide to altruism: Gene-culture coevolution, and the internalization of norms. Journal of Theoretical Biology.

[B30-behavsci-16-00774] Goffman E. (1990). The presentation of self in everyday life.

[B31-behavsci-16-00774] Graham J., Haidt J., Koleva S., Motyl M., Iyer R., Wojcik S. P., Ditto P. H. (2013). Moral foundations theory: The pragmatic validity of moral pluralism. Advances in experimental social psychology.

[B32-behavsci-16-00774] Graham J., Haidt J., Nosek B. A. (2009). Liberals and conservatives rely on different sets of moral foundations. Journal of Personality and Social Psychology.

[B33-behavsci-16-00774] Graham J., Nosek B. A., Haidt J., Iyer R., Koleva S., Ditto P. H. (2011). Mapping the moral domain. Journal of Personality and Social Psychology.

[B34-behavsci-16-00774] Gray K., Waytz A., Young L. (2012). The moral dyad: A fundamental template unifying moral judgment. Psychological Inquiry.

[B35-behavsci-16-00774] Harper C. A., Rhodes D. (2021). Reanalysing the factor structure of the moral foundations questionnaire. British Journal of Social Psychology.

[B36-behavsci-16-00774] Henrich J., Ensminger J., Ensminger J., Henrich J. (2014). Theoretical foundations: The coevolution of social norms, intrinsic motivation, markets, and the institutions of complex societies. Experimenting with social norms: Fairness and punishment in cross-cultural perspective.

[B37-behavsci-16-00774] Henrich J., Heine S. J., Norenzayan A. (2010). Most people are not WEIRD. Nature.

[B38-behavsci-16-00774] Horton J. J., Rand D. G., Zeckhauser R. J. (2011). The online laboratory: Conducting experiments in a real labor market. Experimental Economics.

[B39-behavsci-16-00774] House B. R. (2018). How do social norms influence prosocial development?. Current Opinion in Psychology.

[B40-behavsci-16-00774] Iurino K., Saucier G. (2020). Testing measurement invariance of the Moral Foundations Questionnaire across 27 countries. Assessment.

[B41-behavsci-16-00774] Janoff-Bulman R., Sheikh S., Hepp S. (2009). Proscriptive versus prescriptive morality: Two faces of moral regulation. Journal of Personality and Social Psychology.

[B42-behavsci-16-00774] Kelly D. (2020). Internalized norms and intrinsic motivations: Are normative motivations psychologically primitive?. Emotion Review.

[B43-behavsci-16-00774] Kelly D., Davis T. (2018). Social norms and human normative psychology. Social Philosophy and Policy.

[B44-behavsci-16-00774] Kroneberg C., Heintze I., Mehlkop G. (2010). The interplay of moral norms and instrumental incentives in crime causation. Criminology.

[B45-behavsci-16-00774] Lindström B., Jangard S., Selbing I., Olsson A. (2018). The role of a “common is moral” heuristic in the stability and change of moral norms. Journal of Experimental Psychology: General.

[B46-behavsci-16-00774] Little T. D., Lindenberger U., Nesselroade J. R. (1999). On selecting indicators for multivariate measurement and modeling with latent variables: When “good” indicators are bad and “bad” indicators are good. Psychological Methods.

[B47-behavsci-16-00774] MacKinnon D. P., Fairchild A. J., Fritz M. S. (2007). Mediation analysis. Annual Review of Psychology.

[B48-behavsci-16-00774] Malovicki-Yaffe N., Khan S. A., Paluck E. L. (2023). The social and psychological effects of publicly violating a social norm: A field experiment in the Satmar Jewish community. Frontiers in Social Psychology.

[B49-behavsci-16-00774] McAuliffe K., Blake P. R., Steinbeis N., Warneken F. (2017). The developmental foundations of human fairness. Nature Human Behaviour.

[B50-behavsci-16-00774] McDonald R. I., Crandall C. S. (2015). Social norms and social influence. Current Opinion in Behavioral Sciences.

[B51-behavsci-16-00774] Meng B., Lee M. J., Chua B. L., Han H. (2022). An integrated framework of behavioral reasoning theory, theory of planned behavior, moral norm and emotions for fostering hospitality/tourism employees’ sustainable behaviors. International Journal of Contemporary Hospitality Management.

[B52-behavsci-16-00774] Miles A., Samim Y., Khan S. (2025). Testing the psychological distinctiveness of proscriptive and prescriptive moral norms.

[B53-behavsci-16-00774] Mulder L. B. (2018). When sanctions convey moral norms. European Journal of Law and Economics.

[B54-behavsci-16-00774] Packard C. D., Schultz P. W. (2023). Emotions as the Enforcers of Norms. Emotion Review.

[B55-behavsci-16-00774] Paulus M. (2020). The developmental emergence of morality: A review of current theoretical perspectives. Progress in Brain Research.

[B56-behavsci-16-00774] Pavey L., Sparks P., Churchill S. (2018). Proscriptive vs. prescriptive health recommendations to drink alcohol within recommended limits: Effects on moral norms, reactance, attitudes, intentions and behaviour change. Alcohol and Alcoholism.

[B57-behavsci-16-00774] Rai T. S., Fiske A. P. (2011). Moral psychology is relationship regulation: Moral motives for unity, hierarchy, equality, and proportionality. Psychological Review.

[B58-behavsci-16-00774] R Core Team (2024). R: A language and environment for statistical computing.

[B59-behavsci-16-00774] Rosseel Y. (2012). lavaan: An R package for structural equation modeling. Journal of Statistical Software.

[B60-behavsci-16-00774] Sadek F. H. (2024). Rehashing the moral-conventional distinction: Perceived harm marks the border. Philosophical Psychology.

[B61-behavsci-16-00774] Satorra A., Bentler P. M. (2001). A scaled difference chi-square test statistic for moment structure analysis. Psychometrika.

[B62-behavsci-16-00774] Schein C., Gray K. (2018). The theory of dyadic morality: Reinventing moral judgment by redefining harm. Personality and Social Psychology Review.

[B63-behavsci-16-00774] Schmidt M. F., Rakoczy H., Tomasello M. (2019). Eighteen-month-old infants correct non-conforming actions by others. Infancy.

[B64-behavsci-16-00774] Sheskin M., Santos L. (2012). 23 The evolution of morality: Which aspects of human moral concerns. The Oxford handbook of comparative evolutionary psychology.

[B65-behavsci-16-00774] Smetana J. G., Zelazo P. D. (2013). Moral development: The social domain theory view. The Oxford handbook of developmental psychology (Vol. 1): Body and mind.

[B66-behavsci-16-00774] Smetana J. G., Ball C. L. (2018). Young children’s moral judgments, justifications, and emotion attributions in peer relationship contexts. Child Development.

[B67-behavsci-16-00774] Smetana J. G., Jambon M., Ball C. (2013). The social domain approach to children’s moral and social judgments. Handbook of moral development.

[B68-behavsci-16-00774] Smetana J. G., Yoo H. N. (2022). Development and variations in moral and social conventional judgments: A social domain theory approach. Handbook of moral development.

[B69-behavsci-16-00774] Smith M. K., Masser B. M. (2012). Principles and popularity: The interplay of moral norms and descriptive norms in the context of volunteerism. British Journal of Social Psychology.

[B70-behavsci-16-00774] Sousa P., Holbrook C., Piazza J. (2009). The morality of harm. Cognition.

[B71-behavsci-16-00774] Sullins J., Turner J., Kim J., Barber S. (2024). Investigating the impacts of shame-proneness on students’ state shame, self-regulation, and learning. Education Sciences.

[B72-behavsci-16-00774] Tomasello M. (2018). The normative turn in early moral development. Human Development.

[B73-behavsci-16-00774] Turiel E., Killen M., Helwig C. C., Kagan J. (1987). Morality: Its structure, functions, and vagaries. The emergence of morality in young children.

[B74-behavsci-16-00774] Van Schoelandt C. (2018). Moral accountability and social norms. Social Philosophy and Policy.

[B75-behavsci-16-00774] Yau J., Smetana J. G. (2003). Conceptions of moral, social-conventional, and personal events among Chinese preschoolers in Hong Kong. Child Development.

[B76-behavsci-16-00774] Yucel M., Drell M. B., Jaswal V. K., Vaish A. (2022). Young children do not perceive distributional fairness as a moral norm. Developmental Psychology.

[B77-behavsci-16-00774] Yucel M., Vaish A. (2025). The role of harm salience on children’s and adults’ perceptions of unfairness. Social Cognition.

