# Supplementary material for: Differentiating Social and Moral Norms in Perceived Internalization
Source: Behav Sci (Basel). 2026 May 14;16(5):774. doi: 10.3390/bs16050774 (PMC13203154; doi:10.3390/bs16050774)
Supplement: Supplementary file 1 [file behavsci-16-00774-s001.zip › behavsci-4168746-supplementary.pdf]

Differentiating Social and Moral Norms in Perceived Internalization – Supplementary Materials

Table of Contents

Supplementary Analysis ***2***

Analysis **2**

Primary Models from Main Text 2

Exploratory Models from Main Text - Vignette Valence Models 2

Exploratory Models from Main Text - Sentiment Analysis 3

Preregistered Primary Models 3

Dimension Ratings – Primary Models from Main Text with Covariates 4

Reputation – Primary Models from Main Text without Covariates 4

Reputation – Preregistered Models with Reverse Coded Items 4

Moderation Models 5

Structural Equation Models 5

Results **6**

Primary Models from Main Text 6

Exploratory Models from Main Text - Vignette Valence Models 9

Exploratory Models from Main Text - Sentiment Analysis 10

Preregistered Primary Models 10

Dimension Ratings – Primary Models from Main Text with Covariates 13

Reputation – Primary Models from Main Text without Covariates 14

Reputation – Preregistered Models with Reverse Coded Items 16

Moderation Models 18

Structural Equation Models 20

References **22**

Supplementary Figures & Tables ***22***

Study Materials ***31***

Stimuli **31**

Measures **32**

Supplementary Analysis

Analysis

Primary Models from the Main Text

To confirm that the behavior domains were perceived as corresponding to their hypothesized domains (e.g., fairness behaviors rated as most fair), we ran five multilevel regression models predicting ratings of each behavioral domain dimension rating as a function of behavioral domain for each norm valence (prescriptive and proscriptive) separately, running ten models in total. To explore whether participants' intentions to engage in the behaviors when reputational concerns were salient varied across behaviors, we conducted a series of multilevel regression models predicting the three reputational measures (reputation–general, reputation–public, reputation–common) by behavioral domain. These analyses are identical to those reported in the main text of the paper but include full model results for all model terms. All models controlled for several norm covariate measures (emotional valence, descriptive norms, injunctive norms, moral judgements, punishment–reward evaluations), as well as the random effects for participant and vignette identity. As described in the Method section of the main text, we conducted these planned analyses twice, once for prescriptive behaviors and once for proscriptive behaviors.

Exploratory Models from the Main Text—Vignette Valence Models

We explored differences in vignette valence by conducting three exploratory multilevel regression models. Each model predicted one of our three primary dependent measures (intrinsic motivation, adherence–avoidance importance, reputational sensitivity) and included behavioral domain (harm, conventional, fairness, generosity, purity; reference group = harm), norm valence (prescriptive and proscriptive), and their interaction as predictors. All three models controlled for the random effects of participant and stimuli identity. We compared each of these interaction models to nested models excluding the interaction terms using Likelihood Ratio Tests in order to determine whether including the interaction between behavioral domain and norm valence increased the models' explanatory power.

Exploratory Models from the Main Text—Sentiment Analysis

As an exploratory test of whether participants' behavioral domains definitions varied by behavior, we conducted a sentiment analysis of participants' open-ended definitions. For each definition, we computed continuous word-level sentiment scores using the AFINN lexicon. We first tested for overall differences in sentiment across domains using a one-way ANOVA (which we report in the main text) and then estimated a linear regression model predicting sentiment scores by behavioral domain, with pairwise comparisons conducted using Tukey-adjusted post hoc tests. We also report standardized model-adjusted mean differences (Cohen's *d*) for all pairwise contrasts.

Preregistered Primary Models

We conducted 11 preregistered multilevel regression models. Models 1a-e predicted each behavior dimension rating by behavioral domain and served as a manipulation check to confirm that participants perceived behavioral domains as corresponding to their predetermined categories (e.g., that the fairness behaviors were indeed rated as most fair). Models 2a and 2b predicted adherence (for prescriptive norms) and avoidance (for proscriptive norms) importance by behavioral domain, asking whether participants perceived differences in the importance of adhering to prescriptive behaviors and avoiding proscriptive behaviors. Model 3 predicted intrinsic motivation by behavioral domain to explore whether there were differences in intrinsic motivation between behavioral domains. We conducted three models (models 4a-c) each including behavioral domain, as a predictor to test whether participants' self-reported likelihood of engaging in the behaviors when reputational concerns were salient varied across behaviors. Specifically, model 4a included overall reputational sensitivity (an index of both reputation measures) as the outcome measure, model 4b included reputational sensitivity to common knowledge as the outcome variable, and model 4c included reputational sensitivity to descriptive norms as the outcome variable. Deviating from our preregistration, Models 4a-c included the truncated reputation measures described in the main text (i.e., excluding the two reverse-coded items)—we report the results for these models including all reverse-coded items below (see *Reputation—Preregistered Models with Reverse-Coded Items*).

As initially planned, these models included all data from both prescriptive and proscriptive behaviors. To test our prediction that harm would be perceived as the most

intrinsically motivating, important to adhere to or avoid, and least sensitive to reputational concerns, we set harm as the reference group with which to compare the different behaviors. To test our prediction that conventional behaviors would be perceived as the least intrinsically motivating, important to adhere to or avoid, and most sensitive to reputational concerns, we re-analyzed our models, setting conventional behaviors as the reference group. All models controlled for the random effects of participant and vignette identity.

Dimension Ratings—Primary Models from the Main Text with Covariates

To confirm that the behavioral domains were perceived as corresponding to their hypothesized domains (e.g., fairness behaviors rated as most fair), we ran five multilevel regression models predicting ratings of each behavioral domain dimension as a function of behavioral domain. These analyses are identical to those reported in the main text of the paper but additionally controlled for injunctive norm beliefs, descriptive norm beliefs, emotional valence, moral judgements, and punishment evaluations. All models additionally controlled for the random effects for participant and vignette identity.

Reputation—Primary Models from the Main Text without Covariates

To examine whether participants' intentions to engage in behaviors when reputational concerns were salient varied across behaviors, we conducted a series of multilevel regression models predicting the three reputational measures (reputation–general, reputation–public, reputation–common) by behavioral domain. These analyses are identical to those reported in the main text of the paper but do not control for the norm covariates. All models controlled for the random effects for participant and vignette identity.

Reputation—Preregistered Models with Reverse-Coded Items

As we preregistered, we reverse-coded two of the four items of the reputation measures ("If you could engage in the behavior without anyone knowing or ever finding out, would you be more or less likely to do it?"; "How likely would you be to engage in the behavior if most people you know are not engaging in it?") before combining the four reputation items into two indices—reputation–public and reputation–common. When following this planned scale creation approach, we found that the Cronbach's alpha was over one and negative, suggesting it was inappropriate to combine the reverse-coded items. Nevertheless, we report the results of these

preregistered models here for full transparency. We first ran three multilevel regression models predicting the three reputational measures (reputation–general, reputation–public, reputation–common) by behavioral domain and collapsing across the prescriptive and proscriptive vignettes. To explore whether these results varied by vignette valence, we re-analyzed those three models subsetting our data to only include observations from the prescriptive or proscriptive vignettes.

Moderation Models

We conducted a series of exploratory moderation analyses examining whether perceptions of harm moderated the effect of behavioral domain on our dependent measures. The multilevel harm moderation regression models predicted intrinsic motivation and adherence–avoidance importance by behavioral domain, harm perception ratings, and their interaction. All models controlled for the random effects of participant and vignette ID.

Structural Equation Models

To examine whether harm perception mediated differences in norm internalization between social and moral norm types, we conducted a full structural equation model (SEM) following the two-step procedure of Anderson and Gerbing (1988). This approach first established the psychometric adequacy of the measurement model to ensure that observed differences in latent factor levels were not confounded by measurement artifacts. We modeled norm internalization using two correlated latent factors: intrinsic motivation (intrinsic1, intrinsic2) and norm adherence importance (norm.adhere1, norm.adhere2). All items were standardized prior to analysis. The reputational sensitivity measures were excluded from the latent measurement model because correlations with norm adherence items were near-zero (*r*s = .04–.08 across domains), indicating it captured a conceptually distinct dimension from norm adherence, and more generally, internalization. To stabilize identification with two indicators per factor, residual variances were constrained to equality within each indicator pair—this tau-equivalence constraint is appropriate when items within a pair are parallel measures (Little, Lindenberger, & Nesselroade, 1999).

To ensure that comparisons between participants in the social (conventional, n = 108) and moral (fairness, harm, purity, generosity, n = 427) domains were psychometrically valid, we first conducted a multigroup CFA measurement invariance sequence (configural, metric, scalar) prior to running the full structural model. Model comparisons used the Satorra-Bentler (2001) scaled

chi-square difference test, with effect-size criteria of $\Delta CFI \geq -.010$ and $\Delta RMSEA \geq .015$ denoting meaningful non-invariance (Chen, 2007; Cheung & Rensvold, 2002). The structural model specified norm type (0 = social/conventional, 1 = moral) as a binary observed predictor, harm perception as a latent mediator, and the two internalization latent factors as parallel outcomes. This model estimated three sets of paths simultaneously: (1) the *a* path from norm type to harm perception; (2) the *b* paths from harm perception to each internalization factor; and (3) the c' paths representing the direct effect of norm type on each internalization factor after accounting for harm perception. Indirect effects ($a \times b$) represented the portion of the norm-type–internalization relationship that operates through harm perception. Indirect and total effects were estimated with bootstrapped standard errors (5,000 draws) and bias-corrected accelerated (BCa) 95% confidence intervals (Preacher & Hayes, 2008). Measurement invariance models used maximum likelihood estimation with robust standard errors (MLR) while the full model used maximum likelihood estimation in order to successfully compute the bootstrapped standard errors. All four models used full information maximum likelihood (FIML) to accommodate missing data. Item-level responses were first averaged within-person across the eight behaviors in each participant's assigned domain, yielding one composite score per item per participant (N = 535). We estimated all structural equation models using *lavaan* (Rosseel, 2012). Because the proportion-mediated ratio becomes unstable when direct and indirect effects are of opposite sign or when total effects approach zero (Mackinnon, Fairchild, & Fritz, 2007), the proportion-mediated ratio was calculated descriptively from point estimates rather than using bootstrapped estimates.

Results

Primary Models from the Main Text

Behavioral dimension ratings. We first examined the behavioral dimension ratings and found evidence that behaviors corresponded to their hypothesized domains (see Table 2 for model estimates from all models). Starting with the prescriptive norms, we found that conventional behaviors were rated highest in relevance to social conventions, significantly higher than fairness (B = -6.30, SE = 2.60, p = .016), harm (B = -12.42, SE = 2.64, p < .001), generosity (B = -8.94, SE = 2.63, p < .001), and marginally higher than purity (B = -5.09, SE = 2.62, p = .053). Fairness behaviors were rated as the most fairness-relevant, significantly more so than conventional (B = -33.87, SE = 3.42, p < .001), harm (B = -17.53, SE = 3.46, p < .001), generosity (B = -18.59, SE =

3.45, $p < .001$), or purity (B = -31.54, SE = 3.43, $p < .001$). Similarly, harm behaviors were perceived as significantly more harmful than conventional (B = -43.96, SE = 3.18, $p < .001$), fairness (B = -35.35, SE = 3.16, $p < .001$), generosity (B = -7.65, SE = 3.20, $p = .017$), or purity behaviors (B = -7.46, SE = 3.18, $p = .019$). Prescriptive generosity behaviors were rated as significantly more generous than conventional (B = -34.31, SE = 3.22, $p < .001$), fairness (B = -20.59, SE = 3.20, $p < .001$), and purity behaviors (B = -34.17, SE = 3.23, $p < .001$), but did not differ significantly from harm (B = -0.53, SE = 3.25, $p = .87$). Purity behaviors were rated as the most purity-related, significantly more so than conventional (B = -20.24, SE = 4.01, $p < .001$) and fairness behaviors (B = -11.58, SE = 3.98, $p = .004$), but did not differ significantly from harm (B = 3.78, SE = 4.05, $p = .35$) or generosity behaviors (B = -0.12, SE = 4.04, $p = .98$).

When examining the proscriptive norms, we found that proscriptive conventional behaviors were rated as significantly more of a social convention than harm (B = -13.14, SE = 3.38, $p < .001$), generosity (B = -11.12, SE = 3.37, $p = .001$), and purity (B = -7.62, SE = 3.36, $p = .024$), but did not differ significantly from fairness (B = -2.33, SE = 3.33, $p = .48$). Next, we found proscriptive fairness behaviors were rated as significantly more fairness-related than conventional (B = -22.68, SE = 3.22, $p < .001$), harm (B = -19.66, SE = 3.26, $p < .001$), generosity (B = -7.68, SE = 3.25, $p = .018$), and purity behaviors (B = -43.93, SE = 3.23, $p < .001$). Proscriptive harm behaviors were rated as significantly more harmful than conventional (B = -17.1, SE = 3.39, $p < .001$), fairness (B = -13.29, SE = 3.36, $p < .001$), and generosity (B = -7.89, SE = 3.41, $p = .021$) behaviors, but not purity behaviors (B = -2.09, SE = 3.39, $p = .54$). Proscriptive generosity behaviors were rated as significantly more generous than harm (B = -25.62, SE = 3.43, $p < .001$) and purity behaviors (B = -29.80, SE = 3.41, $p < .001$), marginally more generous than conventional behaviors (B = -6.23, SE = 3.40, $p = .067$), but did not significantly differ from fairness (B = -1.92, SE = 3.37, $p = .57$). Lastly, we found proscriptive purity behaviors were perceived as significantly more purity-related than conventional (B = -19.56, SE = 3.62, $p < .001$), fairness (B = -7.74, SE = 3.60, $p = .032$), and harm behaviors (B = -11.84, SE = 3.66, $p = .001$), but did not differ significantly from generosity behaviors (B = -4.96, SE = 3.65, $p = .17$). Overall, these results indicate that behaviors were perceived to correspond to their hypothesized domain but suggest some conceptual overlap for certain behaviors—i.e., proscriptive harms were perceived as similarly harmful to proscriptive purity behaviors.

Reputation Sensitivity. When reputational concerns were salient, participants reported being less likely to engage in prescriptive harm behaviors than conventional (B = 9.77, SE = 2.21, $p < .001$), fairness (B = 6.20, SE = 2.16, $p = .004$), and purity (B = 8.65, SE = 2.18, $p < .001$) behaviors, but not generosity behaviors (B = -0.003, SE = 2.16, $p = .99$). Participants' intentions to engage in prescriptive conventional behaviors were more sensitive to reputational concerns than harm (B = -9.77, SE = 2.21, $p < .001$) and generosity (B = -9.74, SE = 2.20, $p < .001$), but did not differ significantly from fairness (B = -3.57, SE = 2.12, $p = .09$) or purity behaviors (B = -1.12, SE = 2.13, $p = .60$). Next, turning to the results for the proscriptive norms, we found that participants' intentions to engage in harm behaviors when reputation was salient did not differ significantly from conventional (B = -0.90, SE = 2.28, $p = .69$), fairness (B = 1.48, SE = 2.26, $p = .51$), generosity (B = -0.17, SE = 2.28, $p = .94$), or purity behaviors (B = -0.70, SE = 2.28, $p = .76$). Likewise, participants' intentions to engage in proscriptive conventional behaviors when reputation was salient did not differ significantly from fairness (B = 2.37, SE = 2.24, $p = .29$), harm (B = 0.90, SE = 2.28, $p = .69$), generosity (B = 0.73, SE = 2.26, $p = .75$), or purity (B = 0.20, SE = 2.24, $p = .93$).

We next examined each of the two reputational concern measures individually: reputation–public and reputation–common. When examining differences in behavioral intentions when the behavior would be public and common knowledge, we found that participants reported being less likely to engage in prescriptive harm behaviors than in conventional (B = 10.25, SE = 2.33, $p < .001$), fairness (B = 7.15, SE = 2.28, $p = .002$), and purity behaviors (B = 11.09, SE = 2.30, $p < .001$), but not generosity behaviors (B = 0.02, SE = 2.28, $p = .99$). Participants reported they were more likely to engage in prescriptive conventional behaviors when their behavior would be public and common knowledge as compared to harm (B = -10.25, SE = 2.23, $p < .001$) and generosity (B = -10.23, SE = 2.33, $p < .001$) behaviors—the difference was trending toward significance for fairness (B = -3.09, SE = 2.24, $p = .17$), and did not significantly differ for purity behaviors (B = 0.85, SE = 2.25, $p = .71$). When examining proscriptive behaviors, participants were not more likely to report engaging in harm behaviors than conventional (B = -3.18, SE = 2.30, $p = .17$), fairness (B = -2.06, SE = 2.29, $p = .37$), generosity (B = -1.12, SE = 2.31, $p = .63$), or purity (B = -1.56, SE = 2.30, $p = .50$). Likewise, there were no differences in intentions between proscriptive conventional behaviors and fairness (B = 1.12, SE = 2.26, $p = .62$), harm (B = 3.18, SE = 2.30, $p = .17$), generosity (B = 2.06, SE = 2.29, $p = .37$), or purity (B = 1.62, SE = 2.26, $p = .47$).

Turning to differences in behavioral intentions when behavior was a descriptive norm, we found that participants' intentions were less sensitive to descriptive norms for prescriptive harm behaviors than for conventional (B = 9.39, SE = 2.34, $p < .001$), fairness (B = 5.38, SE = 2.29, $p = .019$), and purity behaviors (B = 6.33, SE = 2.30, $p = .006$)—there was no difference with generosity behaviors (B = 0.008, SE = 2.28, $p = .99$). Behavioral intentions for prescriptive conventional behaviors were more sensitive to descriptive norms than for harm (B = -9.39, SE = 2.34, $p < .001$) and generosity behaviors (B = -9.38, SE = 2.34, $p < .001$); the difference with fairness was trending on significance (B = -4.01, SE = 2.24, $p = .07$), and there was no significant difference for purity (B = -3.06, SE = 2.25, $p = .17$). When looking at proscriptive behaviors, we found no differences in behavioral intention sensitivity to descriptive norms although proscriptive harms were rated as marginally less sensitive than fairness behaviors (B = 5.05, SE = 2.82, $p = .07$). There were no significant differences between proscriptive harms and conventional (B = 1.32, SE = 2.84, $p = .64$), generosity (B = 0.74, SE = 2.85, $p = .80$), or purity behaviors (B =0.23, SE = 2.84, $p = .94$). We also found no difference in behavioral intention sensitivity to descriptive norms for proscriptive conventional behaviors as compared to fairness (B = 3.74, SE = 2.79, $p = .18$), harm (B = -1.32, SE = 2.84, $p = .64$), generosity (B = -0.58, SE = 2.82, $p = .84$), or purity behaviors (B = -1.09, SE = 2.80, $p = .70$).

Exploratory Models from the Main Text—Vignette Valence Models

We found significant interaction effects for adherence–avoidance importance: The effect of behavioral domain varied by norm source such that the difference in importance between prescriptive and proscriptive harm behaviors was significantly smaller than conventional (B = 12.63, SE = 2.29, $p < .001$), fairness (B = 8.88, SE = 2.27, $p < .001$), and purity behaviors (B = -10.15, SE = 2.29, $p < .001$), but did not differ significantly from generosity (B = 2.61, SE = 2.30, $p = .26$). The interaction model explained significantly more variance than the model without the interaction term ($\chi^2(4) = 118.54$, $p < .001$). We also found significant interaction effects between vignette valence and behavioral domain for intrinsic motivation: This time the effect of behavioral domain varied by norm source such that the difference in intrinsic motivation between prescriptive and proscriptive harm behaviors was significantly larger than for conventional (B = -14.85, SE = 2.64, $p < .001$), fairness (B = -27.03, SE = 2.63, $p < .001$), generosity (B = -7.38, SE = 2.66, $p = .006$), and purity behaviors (B = -21.55, SE = 2.65, $p < .001$). The model with the interaction term

explained significantly more variance than the model without it (χ^2 (4) = 132.67, p < .001). We found no evidence of an interaction between behavioral domain and norm source for the reputation sensitivity measure—there was no significant difference in behavioral intentions between the prescriptive and proscriptive harms as compared to conventional (B = 0.36, SE = 2.19, p = .87), fairness (B = -2.29, SE = 2.18, p = .29), or purity behaviors (B = -0.80, SE = 2.20, p = .72). The interaction between norm source and behavioral domain for harm and generosity was trending on significance (B = -4.30, SE = 2.21, p = .052), such that the difference between pre- and proscriptive norms was larger for harm than generosity. The model including the interaction term did not explain significantly more variance in reputational sensitivity than the model without it (χ^2(4) = 6.05, p = .19).

Exploratory Models from the Main Text—Sentiment Analysis

Sentiment scores for the harm behavior domain definitions were significantly more negative than conventional (B = 3.27, SE = 0.19, p < .001, d = -3.27), fairness (B = 3.25, SE = 0.18, p < .001, d = -3.25), generosity (B = 3.80, SE = 0.18, p < .001, d = -3.80), and purity behaviors (B = 3.16, SE = 0.17, p < .001, d = -3.16). When examining the post hoc comparisons between all behavior domains, we found that sentiment scores for generosity behaviors were significantly more positive than fairness (B = 0.55, SE = 0.19, p = .047, d = 0.53), purity (B = 0.64, SE = 0.19, p = .006, d = 0.64), and to a marginally significant extent, conventional behaviors (B = 0.53, SE = 0.20, p = .072, d = 0.53). There were no significant differences in sentiment scores between conventional behaviors and fairness (B = 0.02, SE = 0.20, p = 1, d = 0.02) and purity behaviors (B = 0.11, SE = 0.19, p = .98, d = 0.11), nor between fairness and purity (B = 0.10, SE = 0.19, p = .99, d = 0.10).

Preregistered Primary Models

Domain dimension ratings. We first examined whether the behavioral dimension ratings corresponded to their hypothesized behavior domain, collapsing across vignette valence. Conventional behaviors were rated as significantly more social conventional than harm (B = -12.78, SE = 2.46, p < .001), generosity (B = -10.03, SE = 2.46, p < .001), purity (B = -6.36, SE = 2.44, p < .001), and to a marginal extent, fairness (B = -4.32, SE = 2.42, p = .075). Fairness behaviors were rated as significantly more fairness-related than conventional (B = -28.27, SE =

2.59, $p < .001$), generosity (B = -13.14, SE = 2.61, $p < .001$), harm (B = -18.59, SE = 2.61, $p < .001$), and purity behaviors (B = -37.73, SE = 2.59, $p < .001$). Harm behaviors were perceived as more harm-related than conventional (B = -30.53, SE = 2.67, $p < .001$), fairness (B = -24.33, SE = 2.65, $p < .001$), and generosity behaviors (B = -7.77, SE = 2.69, $p = .004$); the difference between harm and purity was trending on significance (B = -4.78, SE = 2.67, $p = .074$). Participants rated generosity behaviors as significantly more relevant to generosity than conventional (B = -20.27, SE = 2.48, $p < .001$), fairness (B = -11.25, SE = 2.47, $p < .001$), harm (B = -13.08, SE = 2.51, $p < .001$), and purity behaviors (B = -31.98, SE = 2.49, $p < .001$). Lastly, purity behaviors were rated as significantly more purity-related than conventional (B = -19.90, SE = 3.25, $p < .001$) and fairness behaviors (B = -9.66, SE = 3.23, $p = .003$) but not more so than harm (B = -4.03, SE = 3.28, $p = .22$) or generosity (B = -2.54, SE = 3.28, $p = .44$).

Adherence–avoidance importance. Next, we explored whether adherence importance and avoidance importance varied across behavioral domains for prescriptive and proscriptive behaviors. Starting with prescriptive behaviors, we found that harm behavior norms were perceived as significantly more important *to adhere* to than generosity (B = -8.62, SE = 2.78, $p = .002$) and purity behaviors (B = -12.39, SE = 2.76, $p < .001$)—there were no differences in adherence importance between harm and conventional (B = -0.64, SE = 2.76, $p = .82$), or fairness behaviors (B = 3.07, SE = 2.74, $p = .26$). Prescriptive conventional behaviors were rated as more important to adhere to than generosity (B = -7.98, SE = 2.75, $p = .004$) or purity behaviors (B = -11.75, SE = 2.74, $p < .001$) but did not significantly differ from fairness (B = 3.71, SE = 2.71, $p = .17$) or harm (B = 0.64, SE = 2.76, $p = .82$). Turning to the proscriptive behaviors, we found a different pattern of results when exploring which behaviors were perceived as the most important *to avoid*. Harm behaviors were rated as the most important to avoid, more so than conventional (B = -13.27, SE = 2.19, $p < .001$), fairness (B = -5.80, SE = 2.19, $p < .001$), and generosity behaviors (B = -11.23, SE = 2.21, $p < .001$), but did not significantly differ from purity behaviors (B = -2.25, SE = 2.20, $p = .31$). Proscriptive conventional behaviors were rated as less important to avoid than fairness (B = 7.47, SE = 2.16, $p < .001$), harm (B = 13.27, SE = 2.19, $p < .001$), or purity behaviors (B = 11.03, SE = 2.18, $p < .001$), but did not significantly differ from generosity behaviors (B = 2.05, SE = 2.19, $p = .35$).

Intrinsic motivation. We then examined whether participants were the most intrinsically motivated to engage in harm behaviors and the least intrinsically motivated to engage in conventional behaviors. Participants reported significantly higher intrinsic motivation to engage in harm behaviors than conventional (B = -5.74, SE = 2.33, $p = .014$), fairness (B = -8.42, SE = 2.31, $p < .001$), and purity behaviors (B = 6.26, SE = 2.33, $p = .008$); there was no difference in intrinsic motivation between harm and generosity (B = 1.28, SE = 2.34, $p = .59$). Conventional behaviors were rated as significantly less intrinsically motivating than harm (B = 5.74, SE = 2.33, $p = .014$), generosity (B = 7.02, SE = 2.32, $p = .003$), and purity behaviors (B = 11.99, SE = 2.31, $p < .001$), but did not differ significantly from fairness (B = -2.68, SE = 2.29, $p = .24$).

Reputation Sensitivity. When collapsing across vignette valence, we found that participants were significantly less sensitive to general reputational concerns for harm behaviors than conventional (B = 4.24, SE = 1.59, $p = .008$) and purity behaviors (B = 4.46, SE = 1.59, $p = .005$), but were not less sensitive for the harm than fairness (B = 1.31, SE = 1.58, $p = .41$) or generosity behaviors (B = 1.42, SE = 1.60, $p = .38$). Participants were significantly more sensitive to general reputational concerns for conventional behaviors than harm (B = -4.24, SE = 1.59, $p = .008$), but not purity (B = 0.22, SE = 1.58, $p = .89$)—the differences between conventional and fairness (B = -2.93, SE = 1.57, $p = .06$) and generosity (B = -2.82, SE = 1.59, $p = .076$) behaviors were trending on significance. We next examined each reputational sensitivity subscale starting with intentions when the behavior is public and common knowledge. Intentions to engage in harm behaviors when public were significantly lower than purity behaviors (B = 4.63, SE = 1.62, $p = .004$) but did not differ from conventional (B = 2.65, SE = 1.61, $p = .10$), fairness (B = -0.71, SE = 1.60, $p = .66$), or generosity (B = 0.62, SE = 1.62, $p = .70$). Participants' intentions to engage in conventional behaviors were significantly higher than fairness (B = -3.36, SE = 1.59, $p = .035$), but did not otherwise differ from harm (B = -2.65, SE = 1.61, $p = .10$), generosity (B = -2.03, SE = 1.61, $p = .21$), or purity behaviors (B = 1.99, SE = 1.60, $p = .22$). When looking at behavioral intention sensitivity to descriptive norms, we found that participants reported being significantly less likely to engage in harm behaviors than conventional (B = 5.83, SE = 1.92, $p = .002$), purity (B = 4.33, SE = 1.92, $p = .024$), and to a marginally significant extent, fairness behaviors (B = 3.32, SE = 1.90, $p = .08$); there was no difference between harm and generosity (B = 2.21, SE = 1.93, $p = .25$). Participants were significantly more sensitive to descriptive norms in their behavioral intentions for conventional

behaviors than harm (B = -5.83, SE = 1.92, $p = .002$) and to a lesser degree, generosity (B = -3.62, SE = 1.91, $p = .06$), which was trending on significance. There was no difference between conventional and fairness (B = -2.51, SE = 1.89, $p = .18$) or purity (B = -1.49, SE = 1.90, $p = .43$) behaviors.

Dimension Ratings—Primary Models from the Main Text with Covariates

The behavioral dimension ratings supported the hypothesized behavioral domain categorizations even after controlling for norm belief covariates. Starting with the prescriptive norms, we found that conventional behaviors were rated highest in relevance to social conventions, significantly higher than fairness (B = -4.93, SE = 2.28, $p = .031$), harm (B = -18.11, SE = 2.37, $p < .001$), generosity (B = -13.49, SE = 2.37, $p < .001$), and purity (B = -5.64, SE = 2.29, $p = .014$). Fairness behaviors were rated as the most fairness-relevant, significantly more so than conventional (B = -32.45, SE = 3.31, $p < .001$), harm (B = -23.63, SE = 3.37, $p < .001$), generosity (B = -24.27, SE = 3.37, $p < .001$), and purity (B = -31.69, SE = 3.30 $p < .001$). Similarly, harm behaviors were perceived as significantly more harmful than conventional (B = -35.43, SE = 3.03, $p < .001$), fairness (B = -27.49, SE = 2.98, $p < .001$), generosity (B = -7.14, SE = 2.98, $p = .017$), but not purity behaviors (B = -0.31, SE = 3.00, $p = .92$). Prescriptive generosity behaviors were rated as significantly more generous than conventional (B = -25.58, SE = 3,21, $p < .001$), fairness (B = -13.52, SE = 3.15, $p < .001$), and purity behaviors (B = -27.53, SE = 3.18, $p < .001$), but did not differ from harm behaviors (B = -0.85, SE = 3.15, $p = .79$). Purity behaviors were rated as the most purity-related, significantly more so than conventional (B = -19.82, SE = 3.94, $p < .001$) and fairness behaviors (B = -11.05, SE = 3.91, $p = .005$), but did not differ significantly from harm (B = -0.09, SE = 4.00, $p = .98$) or generosity behaviors (B = -3.83, SE = 4.00, $p = .34$).

Next, when examining the proscriptive norms, we found that proscriptive conventional behaviors were rated as significantly more of a social convention than harm (B = -11.30, SE = 3.33, $p < .001$), generosity (B = -8.67, SE = 3.37, $p = .01$), and purity (B = -7.77, SE = 3.33, $p = .02$), but did not differ significantly from fairness (B = -0.64, SE = 3.33, $p = .85$). Proscriptive fairness behaviors were rated as significantly more fairness-related than conventional (B = -20.68, SE = 3.24, $p < .001$), harm (B = -20.13, SE = 3.28, $p < .001$), generosity (B = -6.99, SE = 3.25, $p = .03$), or purity behaviors (B = -42.93, SE = 3.25, $p < .001$). Proscriptive harm behaviors were rated as significantly more harmful than conventional (B = -14.76, SE = 3.40, $p < .001$), fairness

(B = -12.40, SE = 3.37, $p < .001$), and generosity (B = -7.01, SE = 3.40, $p = .04$) behaviors, but not purity behaviors (B = -0.75, SE = 3.40, $p = .83$). Proscriptive generosity behaviors were rated as significantly more generous than harm (B = -27.19, SE = 3.46, $p < .001$) and purity behaviors (B = -30.18, SE = 3.44, $p < .001$), but did not significantly differ from conventional (B = -5.75, SE = 3.43, $p = .09$) or fairness behaviors (B = -3.10, SE = 3.40, $p = .36$). Lastly, proscriptive purity behaviors were perceived as significantly more purity-related than conventional (B = -18.76, SE = 3.62, $p < .001$), fairness (B = -8.73, SE = 3.61, $p = .016$), and harm behaviors (B = -13.93, SE = 3.67, $p < .001$), but did not differ significantly from generosity behaviors (B = -5.90, SE = 3.65, $p = .11$). Overall, these results indicate that the behaviors were perceived to correspond to their hypothesized domain but suggest some conceptual overlap for certain behaviors—i.e., proscriptive harms were perceived as similarly relevant to harm as proscriptive purity behaviors.

Reputation—Primary Models from the Main Text without Covariates

We next examined how reputational concerns influenced behavioral intentions across behavioral domains for prescriptive and proscriptive behaviors individually, without the inclusion of the norming covariate measures. When reputational concerns were salient, participants reported they were no less likely to engage in the prescriptive harm behaviors than conventional (B = 4.42, SE = 2.36, $p = .06$), fairness (B = 0.16, SE = 2.34, $p = .95$), generosity (B = -0.74, SE = 2.37, $p = .76$), or purity behaviors (B = 4.06, SE = 2.36, $p = .09$), although the difference was trending on significance for the conventional and purity. Participants intentions to engage in prescriptive conventional behaviors were more sensitive to reputational concerns than generosity behaviors (B = -4.42, SE = 2.36, $p = .029$), and marginally more sensitive than fairness (B = -4.26, SE = 2.32, $p = .066$) and harm (B = -5.16, SE = 2.35, $p = .061$), but did not differ significantly from purity (B = -0.36, SE = 2.34, $p = .88$). When examining proscriptive behaviors, we found that proscriptive harm behaviors were rated as marginally less sensitive to reputational concerns than purity behaviors (B = 4.86, SE = 2.64, $p = .066$), but otherwise did not differ significantly compared to conventional (B = 4.06, SE = 2.64, $p = .124$), fairness (B = 2.45, SE = 2.62, $p = .35$), and generosity behaviors (B = 3.57, SE = 2.65, $p = .18$). Proscriptive conventional behaviors were not perceived as more sensitive to reputational concerns than fairness (B = -1.61, SE = 2.59, $p = .54$), harm (B = -0.49, SE = 2.63, $p = .12$), generosity (B = -4.06, SE = 2.64, $p = .85$), or purity behaviors (B = 0.80, SE = 2.62, $p = .76$).

We then examined each of the two reputational concern measures individually: reputation–public and reputation–common. When examining differences in behavioral intentions when prescriptive behaviors would be public and common knowledge, we found that participants reported they would be less likely to engage in harm behaviors than purity behaviors (B = 5.87, SE = 2.49, p = .019) but were as likely to engage in harm behaviors as conventional (B = 4.16, SE = 2.48, p = .095), fairness (B = 0.39, SE = 2.47, p = .87), or generosity behaviors (B = -0.79, SE = 2.50, p = .75). Participants reported they were more likely to engage in prescriptive conventional behaviors than prescriptive generosity behaviors (B = -4.16, SE = 2.48, p = .046), but not fairness (B = -3.76, SE = 2.44, p = .123), harm (B = -4.96, SE = 2.48, p = .095), or purity behaviors (B = 1.71, SE = 2.47, p = .49). Turning to proscriptive behaviors, participants intentions to engage in harm behaviors were no less sensitive to common knowledge than conventional (B = 1.14, SE = 2.68, p = .67), fairness (B = -1.82, SE = 2.66, p = .49), generosity (B = 2.04, SE = 2.70, p = .45), or purity (B = 3.39, SE = 2.69, p = .21) behaviors. Similarly, participants were no more sensitive to common knowledge for proscriptive conventional behaviors than fairness (B = -2.96, SE = 2.64, p = .26), harm (B = 0.89, SE = 2.68, p = .67), generosity (B = -1.14, SE = 2.68, p = .74), or purity (B = 2.26, SE = 2.66, p = .39) behaviors.

Turning to differences in behavioral intentions when prescriptive behaviors were descriptive norms, we found that participants' intentions were no less sensitive to descriptive norms for harm behaviors than fairness (B = -0.08, SE = 2.43, p = .97), generosity (B = -0.68, SE = 2.47, p = .78), or purity behaviors (B = 2.25, SE = 2.45, p = .36), although there was a difference trending on significance between harm and conventional behaviors (B = 4.68, SE = 2.49, p = .057). Participants' intentions were less sensitive to descriptive norms for conventional than fairness (B = -4.76, SE = 2.41, p = .049), generosity (B = -4.68, SE = 2.45, p = .029), and to a marginal extent, harm behaviors (B = 5.36, SE = 2.44, p = .057)—the difference between conventional and purity behaviors was not significant (B = -2.43, SE = 2.43, p = .318). For proscriptive behaviors, participants reported they would be less likely to engage in harm behaviors when there was a common descriptive norm than conventional (B = 6.98, SE = 3.11, p = .026), fairness (B = 6.72, SE = 3.09, p = .03), and purity behaviors (B = 6.45, SE = 3.12, p = .039). There was no significant difference between harm and generosity behaviors (B = 5.10, SE = 3.14, p = .11). When comparing proscriptive conventional behaviors, we found that participants' intentions for conventional behaviors were more sensitive to descriptive norms than

harm (B = -6.97, SE = 3.11, p = .026), but did not differ significantly from fairness (B = -0.26, SE = 3.06, p = .93), generosity (B = -1.87, SE = 3.11, p = .55), or purity behaviors (B = -0.53, SE = 3.09, p = .87).

Reputation—Preregistered Models with Reverse-Coded Items

We next examined whether participants' intentions to engage in behaviors were influenced by reputational concerns to different extents across the five behaviors when using all of our planned items, including the two reverse-coded items. Intentions to engage in harm behaviors were significantly less sensitive to reputational concerns than conventional (B = 2.99, SE = 0.60, p < .001) and fairness behaviors (B = 2.08, SE = 0.59, p < .001), and marginally less sensitive than purity behaviors (B = 1.18, SE = 0.60, p = .051); there was no difference in intentions for harm and generosity behaviors (B = 0.45, SE = 0.61, p = .46). Intentions to engage in conventional behaviors were more sensitive to reputational concerns than harm (B = -2.99, SE = 0.60, p < .001), generosity (B = -2.55, SE = 0.60, p < .001), and purity behaviors (B = -1.82, SE = 0.59, p = .003), but not fairness behaviors (B = -0.92, SE = 0.59, p = .12).

We now individually examine two reputational concern measures: reputation–public and reputation–common. First, we explored behavioral intentions when the behavior was publicly observable. Participants were neither more nor less likely to report they would engage in harm behaviors when public than conventional (B = 1.17, SE = 0.72, p = .11), fairness (B = -0.50, SE = 0.71, p = .48), generosity (B = -0.32, SE = 0.72, p = .65), or purity (B = 0.92, SE = 0.72, p = .20) behaviors. Participants were significantly more likely to report they would engage in conventional behaviors when public than fairness (B = -1.67, SE = 0.71, p = .019) and generosity behaviors (B = -1.49, SE = 0.72, p = .038), but not harm (B = -1.17, SE = 0.72, p = .11) or purity (B = -0.24, SE = 0.71, p = .73). Second, we examined whether behavioral intentions when the behavior was a descriptive norm varied by behavioral domain. Participants' were less likely to report they would engage in harm behaviors than conventional (B = 4.83, SE =1.09 , p < .001) and fairness behaviors (B = 4.66, SE = 1.08, p < .001); there was no difference between harm and generosity (B = 1.23, SE = 1.09, p = .26) or harm and purity (B = 1.44, SE = 1.09, p = .19). Participants were significantly more likely to report they would engage in conventional behaviors when there was a descriptive norm than harm (B = -4.83, SE = 1.09, p < .001),

generosity (B = -3.60, SE = 1.09, $p < .001$), and purity behaviors (B = -3.39, SE = 1.08, $p = .002$), but did not differ significantly from fairness (B = -0.17, SE = 1.07, $p = .87$).

Next, we conducted the same analyses for both prescriptive and proscriptive norms individually to better understand how the behaviors varied across norm valence. When reputational concerns were salient, participants reported they were significantly less likely to engage in prescriptive harm behaviors than prescriptive conventional (B = 5.29, SE = 0.92, $p < .001$), fairness (B = 5.31, SE = 0.92, $p < .001$), generosity (B = 1.91, SE = 0.93, $p = .04$), and purity (B = 3.19, SE = 0.93, $p < .001$) behaviors. Participants were more likely to report they would engage in prescriptive conventional behaviors than the prescriptive harm (B = 5.29, SE = 0.92, $p < .001$), generosity (B = -3.38, SE = 0.92, $p < .001$), and purity (B = -2.09, SE = 0.92, $p = .02$), but not fairness (B = 0.02, SE = 0.91, $p = .98$). Turning to proscriptive behaviors, we found that participants' behavioral intentions were no less sensitive to reputational concern for harm than conventional (B = 0.70, SE = 0.73, $p = .34$), fairness (B = -1.16, SE = 0.73, $p = .11$), generosity (B = -1.01, SE = 0.74, $p = .17$), or purity (B = -0.83, SE = 0.73, $p = .26$). Participants were significantly less sensitive to reputational concerns in their intentions for proscriptive conventional behaviors than fairness (B = -1.86, SE = 0.72, $p = .01$), generosity (B = -1.71, SE = 0.73, $p = .02$), and purity behaviors (B = -1.53, SE = 0.73, $p = .04$), but not harm (B = -0.70, SE = 0.73, $p = .34$).

We next examined each of the reputational concern subscales starting with reputational concern–public, assessing intentions when the behavior is public and common knowledge. Intentions for prescriptive harm behaviors were significantly less sensitive to reputational concerns than conventional (B = 4.45, SE = 0.97, $p < .001$), fairness (B = 4.29, SE = 0.97, $p < .001$), purity (B = 4.27, SE = 0.98, $p < .001$), and to a marginal extent, generosity (B = 1.87, SE = 0.98, $p = .06$). Intentions for prescriptive conventional behaviors were significantly more sensitive to harm (B = -4.45, SE = 0.97, $p < .001$), and generosity (B = -2.59, SE = 0.97, $p = .008$) behaviors but did not differ from fairness (B = -0.17, SE = 0.96, $p = .86$) or purity (B = -0.18, SE = 0.97, $p = .85$). Participants' intentions were more sensitive to the behavior being public for proscriptive harms than fairness (B = -5.29, SE = 1.33, $p < .001$), and to a marginal extent, generosity (B = -2.51, SE = 1.35, $p = .063$) and purity (B = -2.43, SE = 1.34, $p = .07$); there was no difference between harm and conventional behaviors (B = -2.12, SE = 1.34, $p = .11$). Participants intentions were more sensitive to the behavior being public for proscriptive

conventional behaviors than fairness behaviors (B = -3.16, SE = 1.32, $p = .02$), but did not differ significantly for harm (B = 2.12, SE = 1.34, $p = .11$), generosity (B = -0.39, SE = 1.34, $p = .77$), or purity behaviors (B = -0.31, SE = 1.33, $p = .82$).

Turning to the reputational concern–common measure assessing intentions when the behavior is a descriptive norm. We found that participants' intentions for the prescriptive harms were less sensitive to descriptive norms than prescriptive conventional (B = 6.13, SE = 1.22, $p < .001$), fairness (B = 6.34, SE = 1.21, $p < .001$), and marginally less sensitive than purity (B = 2.11, SE = 1.22, $p = .085$); they did not differ from generosity behaviors (B = 1.96, SE = 1.23, $p = .11$). Participants' intentions for prescriptive conventional behaviors were significantly more sensitive to descriptive norms than harm (B = -6.13, SE = 1.22, $p < .001$), generosity (B = -4.16, SE = 1.22, $p < .001$), and purity (B = -4.02, SE = 1.21, $p < .001$), but not fairness (B = 0.21, SE = 1.19, $p = .86$). Proscriptive harms were perceived as less sensitive to descriptive norms than proscriptive conventional (B = 3.53, SE = 1.31, $p = .007$) and fairness (B = 2.97, SE = 1.30, $p = .02$) behaviors, but did not differ from generosity (B = 0.49, SE = 1.32, $p = .71$) or purity behaviors (B = 0.77, SE = 1.31, $p = .56$). Lastly, participants were more sensitive to descriptive norms in their intentions for the proscriptive conventional behaviors than proscriptive harm (B = -3.53, SE = 1.31, $p = .007$), generosity (B = -3.04, SE = 1.31, $p = .02$), and purity (B = -2.75, SE = 1.30, $p = .035$) behaviors; there was no difference with fairness (B = -0.55, SE = 1.29, $p = .67$).

Moderation Models

Using a series of multilevel regression models, we first tested whether perceptions of harm moderated the effect of behavioral domain on intrinsic motivation and adherence–avoidance importance for prescriptive and proscriptive behaviors using multilevel regression models. For prescriptive behaviors, we found marginally significant interaction effects of harm perceptions and behavioral domain on intrinsic motivation: harm and conventional behaviors (B = -0.15, SE = 0.08, $p = .051$) and harm and generosity (B = -0.14, SE = .08, $p = .078$). The interaction terms with harm and fairness (B = -0.11, SE = 0.08, $p = .16$) and harm and purity (B = -0.02, SE = 0.08, $p = .75$) were not significant. Although harm perceptions showed a strong main effect on intrinsic motivation (B = 0.25, SE = 0.06, $p < .001$), such that behaviors participants perceived as more harmful were viewed as more intrinsically motivating, the inclusion of the interaction terms did not significantly improve model fit relative to a model only including the main effect of

behavioral domain (χ^2 (4) = 7.49, p = .11). This suggests that while harm perceptions influenced intrinsic motivation, perceptions of harm did not significantly moderate differences in intrinsic motivation between the prescriptive behavioral domains.

For adherence importance, we found several significant interaction effects between harm perceptions and behavioral domain—harm perceptions increased adherence importance for the harm behaviors to a greater degree than for conventional (B = -0.22, SE = 0.08, p = .006), fairness (B = -0.22, SE = 0.08, p = .005), and generosity behaviors (B = -0.30, SE = 0.08, $p <$ .001), but not purity behaviors (B = -0.13, SE = 0.08, p = .11). The main effect of harm perception was also significant (B = 0.26, SE = 0.07, $p <$ .001), such that higher harm perceptions were associated with greater adherence importance. Overall, the model with the interaction term explained significantly more variance than a model without it (χ^2 (4) = 15.34, p = .004), suggesting that harm perceptions moderated the differences between behaviors in adherence importance.

Next, we examined the moderating effect of harm perceptions for proscriptive behaviors. All interaction effects between harm perceptions and the behavioral domain comparisons were significant such that harm perceptions increased intrinsic motivation to a greater extent for harm behaviors than conventional (B = -0.14, SE = 0.06, p = .026), fairness (B = -0.21, SE = 0.06, p = .001), generosity (B = -0.16, SE = 0.06, p = .016), and purity behaviors (B = -0.20, SE = 0.06, p = .002). The main effect of harm perceptions was also significant (B = 0.17, SE = 0.05, $p <$ .001), indicating that higher harm perceptions were associated with intrinsic motivation to engage in the behaviors. The model with the interaction term explained significantly more variance than the model without it (χ^2 (4) = 12.89, p = .012), suggesting that harm perceptions attenuated the observed differences between proscriptive behaviors in intrinsic motivation.
The interaction effect between harm perception and behavioral domain on avoidance importance was significant when comparing harm with conventional behaviors (B = 0.17, SE = 0.05, p = .001), indicating that increases in perceived harm were associated with greater increases in avoidance importance for conventional behaviors than for harm behaviors. We also observed a marginal interaction between harm and fairness (B = -0.10, SE = 0.05, p = .07). The interactions terms comparing harm with generosity (B = -0.08, SE = 0.05, p = .16) and purity (B = 0.07, SE = 0.05, p = .16) were not statistically significant. This time the main effect of harm perception was not significant (B = 0.03, SE = 0.04, p = .49), suggesting that beliefs about avoiding proscriptive

behaviors were not sensitive to how harmful they were perceived to be. The model with the interaction between harm perceptions and behavioral domain explained significantly more variance than a model without that term (χ^2 (4) = 38.04, $p < .001$), suggesting that harm perceptions moderated domain-level differences in avoidance importance.

Structural Equation Models

Configural invariance was supported (CFI = .996, RMSEA = .050, SRMR = .018, p = .140), confirming that the two-factor structure of internalization held equivalently in both groups. Metric invariance was also fully supported: constraining factor loadings to be equal across groups did not significantly worsen model fit ($\Delta\chi^2(2) = 3.58$, p = .167) and produced negligible changes in all model fit indices well within accepted thresholds (ΔCFI = −.001, ΔRMSEA = −.001, ΔSRMR = .004). The AIC favored the metric model (4673.7) over the configural model (4675.2), supporting the more parsimonious assumption of equal loadings across groups. These results confirm that intrinsic motivation and norm adherence importance are measured with equivalent construct validity for participants in both the social and moral norms domains, supporting direct comparisons of latent factor levels across groups. Full scalar invariance was not supported ($\Delta\chi^2(2) = 8.23$, p = .016), though effect-size changes remained modest (ΔCFI = −.004, ΔRMSEA = +.011) and below conventional thresholds. Score tests (i.e., Lagrange Multiplier tests; Breusch & Pagan, 1980) identified the source of non-invariance as the intercepts of the two norm adherence items: participants in the moral norm domains showed lower baseline responses on these items independent of their latent adherence level. Intrinsic motivation item intercepts were fully invariant (χ^2s ≤ 0.40, ps ≥ .529). Partial scalar invariance could not be distinguished from the metric model given the two-indicator-per-factor design, so structural analyses were conducted under the metric invariance model. See Table S7 for a table of fit indices for each of the three invariance models.

The full structural SEM provided adequate fit to the data, $\chi^2(8) = 67.10$, CFI = .962, RMSEA = .118, 90% CI [.092, .144], SRMR = .068 (see Figure S3 for the path diagram of this model). Specifically, the CFI and SRMR values indicated acceptable fit while the elevated RMSEA reflected model parsimony constraints inherent in the binary norm-type predictor design (e.g., social vs moral), which collapsed four distinct moral domains into a single group. Table S8 presents all parameter estimates with bootstrapped 95% BCa confidence intervals. For the path between norm type (social, moral) and harm perceptions, we found that participants in the moral

norm group rated behaviors as significantly more harmful than those in the social norm group (B = 0.194, SE = 0.024, z = 8.24, p < .001, β = .080, 95% BCa CI [0.154, 0.248]). BCa and percentile confidence intervals were nearly identical, confirming estimate stability across bootstrap draws. When examining the paths from harm perception to the internalization factors, we found that higher harm perceptions were significantly associated with greater intrinsic motivation (B = 0.305, SE = 0.045, p < .001, β = .332, 95% BCa CI [0.217, 0.393]) and greater norm adherence importance (B = 0.262, SE = 0.045, p < .001, β = .273, 95% BCa CI [0.174, 0.355]), such that a one standard deviation increase in harm perception was associated with approximately one-third (one-quarter) of a standard deviation increase in intrinsic motivation (adherence–avoidance importance). Next, examining the direct effect of norm type on internalization, we found that after accounting for harm perception, norm type had no significant direct effect on intrinsic motivation (B = 0.009, SE = 0.106, p = .932, β = .004, 95% BCa CI [−0.194, 0.209]) or on norm adherence importance (B = −0.093, SE = 0.105, p = .375, β = −.040, 95% BCa CI [−0.302, 0.117]). Both confidence intervals spanned zero, indicating that norm type had no independent effect on internalization once harm perception was accounted for.

The indirect effect of norm type on intrinsic motivation through harm perception was significant and positive ($a \times b1$ = 0.059, SE = 0.011, p < .001, β = .064, 95% BCa CI [0.040, 0.085]). The indirect effect on norm adherence importance was also significant ($a \times b2$ = 0.051, SE = 0.011, p < .001, β = .053, 95% BCa CI [0.033, 0.076]). Both indirect effects were estimated with confidence intervals that did not approach zero, and BCa and percentile CIs were substantively identical, confirming bootstrap stability. Lastly, the total effects of norm type on both internalization outcomes were non-significant (intrinsic motivation: B = 0.068, p = .505; norm adherence: B = −0.043, p = .674), indicating that collapsing four diverse moral domains into a single binary group attenuated the overall group difference. Despite this, harm perception accounted for 86.8% of the total effect on intrinsic motivation (indirect effect / total effect = 0.059 / 0.068). For norm adherence, the direct ($c'2$ = −0.093) and indirect effects (0.051) were of opposite sign, producing a suppression pattern in which harm perception and norm type exerted opposing influences on norm adherence, yielding a near-zero total effect. The proportion mediated is not interpretable in the conventional sense under suppression (MacKinnon, Fairchild, & Fritz, 2007).

References

Breusch, T. S., & Pagan, A. R. (1980). The Lagrange multiplier test and its applications to model specification in econometrics. *The Review of Economic Studies, 47*(1),

Chen, F. F. (2007). Sensitivity of goodness of fit indexes to lack of measurement invariance. *Structural Equation Modeling: A Multidisciplinary Journal, 14(*3), 464-504.

Cheung, G. W., & Rensvold, R. B. (2002). Evaluating goodness-of-fit indexes for testing measurement invariance. *Structural Equation Modeling, 9*(2), 233-255.

Little, T. D., Lindenberger, U., & Nesselroade, J. R. (1999). On selecting indicators for multivariate measurement and modeling with latent variables: When" good" indicators are bad and" bad" indicators are good. *Psychological Methods*, *4*(2), 192.

MacKinnon, D. P., Fairchild, A. J., & Fritz, M. S. (2007). Mediation analysis. *Annual Review of Psychology, 58*(1), 593-614.

Satorra, A., & Bentler, P. M. (2001). A scaled difference chi-square test statistic for moment structure analysis. *Psychometrika, 66*(4), 507-514.

Supplementary Figures and Tables

Table S1. Table showing averages and standard deviations for the five dimension ratings for each behavioral domain and the Pearson's correlation coefficient across dimension ratings.

Behavioral Domain	Conventional Avg	Conventional SD	Fairness Avg	Fairness SD	Harm Avg	Harm SD	Purity Avg	Purity SD	Generosity Avg	Generosity SD
Conventional	71.875	29.14674	42.38889	32.8835	39.40162	33.35903	28.46991	30.06677	49.29282	33.31922
Fairness	67.55919	29.56896	70.71477	30.41988	45.59188	32.66587	38.66065	32.11869	58.28749	32.58456
Generosity	61.85	31.08079	57.525	34.36305	62.1619	33.43123	45.82619	35.18612	69.55952	32.72501
Harm	59.09375	32.93444	52.06971	34.42818	69.93269	31.95775	44.33894	35.05499	56.48438	36.89129
Purity	65.51986	31.67355	32.92991	32.74712	65.15537	34.07948	48.36916	37.40794	37.57477	34.14385

Correlation Matrix

	Conventional	Fairness	Harm	Purity	Generosity
Conventional	–				
Fairness	0.3	–			
Harm	0.25	0.24	–		
Purity	0.15	0.4	0.32	–	
Generosity	0.25	0.55	0.33	0.38	–

Table S2 Table showing averages and standard deviations for the five dimension ratings for each behavioral domain separated by norm valence.

Behavioral Domain	Norm Valence	Conventional Avg	Conventional SD	Fairness Avg	Fairness SD	Harm Avg	Harm SD	Purity Avg	Purity SD	Generosity Avg	Generosity SD
Conventional	Proscriptive	62.88	32.42	45.14	32.43	39.97	32.99	28.25	30.33	54.22	32.03
Conventional	Prescriptive	80.87	22.09	39.64	33.14	38.83	33.76	28.69	29.84	44.37	33.89
Fairness	Proscriptive	60.55	32.01	67.82	31.63	43.78	32.27	40.07	32.93	58.53	33.87
Fairness	Prescriptive	74.58	25.04	73.62	28.90	47.41	33.00	37.25	31.26	58.04	31.28
Generosity	Proscriptive	51.77	33.10	60.14	34.78	49.18	33.59	42.85	34.26	60.45	34.31
Generosity	Prescriptive	71.93	25.20	54.91	33.78	75.14	27.78	48.80	35.88	78.67	28.29
Harm	Proscriptive	49.75	34.84	48.16	34.87	57.07	34.74	35.97	32.42	34.83	32.26
Harm	Prescriptive	68.44	27.98	55.98	33.57	82.80	22.50	52.71	35.63	78.14	27.28
Pruity	Proscriptive	55.26	32.85	23.89	26.56	54.98	35.47	47.81	37.26	30.65	31.26
Purity	Prescriptive	75.78	26.81	41.97	35.75	75.33	29.33	48.93	37.59	44.50	35.51

Table S3. Model parameters and estimates. Models 1a-4c were our primary analyses. All models included the random effects of participant and behavior ID and controlled for covariates (emotional valence, descriptive norms, injunctive norms, punishment-reward evaluations, and moral judgements). For models 1a-e we set the reference group corresponding to the relevant dimension comparisons (e.g., fairness was the reference group when comparing the extent to which the behaviors pertained to fairness), while for models 2-4c, harm was set as the reference group.

Model	Results - Prescriptive	Results - Proscriptive
1a. Conventional (continuous: 0 - 100) ~ Behavioral domain	Behavior: Conv – Fair: B = -6.29*<br>Behavior: Conv – Harm: B = -12.42***<br>Behavior: Conv – Gen: B = -8.94***<br>Behavior: Conv – Purity: B = -5.09†	Behavior: Conv – Fair: B = -2.33<br>Behavior: Conv – Harm: B = -13.14***<br>Behavior: Conv – Gen: B = -11.12**<br>Behavior: Conv – Purity: B = -7.62*
1b. Fairness (continuous: 0 -100) ~ Behavioral domain	Behavior: Fair – Conv: B = -33.87***<br>Behavior: Fair – Harm: B = -17.53***	Behavior: Fair – Conv: B = -22.68***<br>Behavior: Fair – Harm: B = -19.66***

	Behavior: Fair – Gen: B = -18.59***<br>Behavior: Fair – Purity: B = -31.54***	Behavior: Fair – Gen: B = -7.68*<br>Behavior: Fair – Purity: B = -43.93***
1c. Harm (continuous: 0 - 100) ~ Behavioral domain	Behavior: Harm – Conv: B = -43.96***<br>Behavior: Harm – Fair: B = -35.35***<br>Behavior: Harm – Gen: B = -7.65*<br>Behavior: Harm – Purity: B = -7.46*	Behavior: Harm – Conv: B = -17.1***<br>Behavior: Harm – Fair: B = -13.29***<br>Behavior: Harm – Gen: B = -7.89*<br>Behavior: Harm – Purity: B = -2.09
1d. Generosity (continuous: 0 - 100) ~ Behavioral domain	Behavior: Gen – Conv: B = -34.31***<br>Behavior: Gen – Fair: B = -20.59***<br>Behavior: Gen – Harm: B = -0.53<br>Behavior: Gen – Purity: B = -34.17***	Behavior: Gen – Conv: B = -6.23†<br>Behavior: Gen – Fair: B = -1.92<br>Behavior: Gen – Harm: B = -25.62***<br>Behavior: Gen – Purity: B = -29.80***
1e. Purity (continuous: 0 - 100) ~ Behavioral domain	Behavior: Purity – Conv: B = -20.24***<br>Behavior: Purity – Fair: B = -11.58**<br>Behavior: Purity – Harm: B = 3.78<br>Behavior: Purity – Gen: B = -0.12	Behavior: Purity – Conv: B = -19.56***<br>Behavior: Purity – Fair: B = -7.74*<br>Behavior: Purity – Harm: B = -11.84**<br>Behavior: Purity – Gen: B = -4.96
2a. Importance–Adhere (continuous: 0 - 100) ~ Behavioral domain	Behavior: Harm – Conv: B = 1.23<br>Behavior: Harm – Fair: B = 5.05†<br>Behavior: Harm – Gen: B = -7.97**<br>Behavior: Harm – Purity: B = -10.87***	
2b. Importance–Avoid (continuous: 0 - 100) ~ Behavioral domain		Behavior: Harm – Conv: B = -10.82***<br>Behavior: Harm – Fair: B = -5.48*<br>Behavior: Harm – Gen: B = -10.57***<br>Behavior: Harm – Purity: B = -0.18
3. Intrinsic motivation (continuous: 0 - 100) ~ Behavioral domain	Behavior: Harm – Conv: B = -9.50***<br>Behavior: Harm – Fair: B = -18.02***<br>Behavior: Harm – Gen: B = -2.26<br>Behavior: Harm – Purity: B = -1.12	Behavior: Harm – Conv: B = -1.73<br>Behavior: Harm – Fair: B = 3.20<br>Behavior: Harm – Gen: B = 2.41<br>Behavior: Harm – Purity: B = 13.61***

4a. Reputation–General	Behavior: Harm – Conv: B = 9.77***<br>Behavior: Harm – Fair: B = 6.20**<br>Behavior: Harm – Gen: B = 0.03<br>Behavior: Harm – Purity: B = 8.65***	Behavior: Harm – Conv: B = -0.89<br>Behavior: Harm – Fair: B = 1.48<br>Behavior: Harm – Gen: B = -0.17<br>Behavior: Harm – Purity: B = -0.69
4b. Reputation–Common Knowledge	Behavior: Harm – Conv: B = 10.20***<br>Behavior: Harm – Fair: B = 7.10**<br>Behavior: Harm – Gen: B = 0.03<br>Behavior: Harm – Purity: B = 11.06***	Behavior: Harm – Conv: B = -3.18<br>Behavior: Harm – Fair: B = -2.06<br>Behavior: Harm – Gen: B = -1.12<br>Behavior: Harm – Purity: B = -1.56
4c. Reputation–Descriptive Norms	Behavior: Harm – Conv: B = -9.39***<br>Behavior: Harm – Fair: B = 5.38*<br>Behavior: Harm – Gen: B = 0.01<br>Behavior: Harm – Purity: B = 6.33**	Behavior: Harm – Conv: B = 1.32<br>Behavior: Harm – Fair: B = 5.05‡<br>Behavior: Harm – Gen: B = 0.74<br>Behavior: Harm – Purity: B = 0.23

Table S4. Model table with path estimates for the harm perception mediation model comparing harm with conventional behaviors.

Effect	Estimate	SE	z	p	CI	Std Est
c	-1.264	2.573	-0.49	0.623	[-6.31, 3.78]	-0.020
a	30.478	2.769	11.01	< .001	[25.05, 35.91]	0.423
b	0.230	0.037	6.28	< .001	[0.16, 0.30]	0.258
ab	7.008	1.254	5.59	< .001	[4.55, 9.47]	0.109
Total effect	5.744	2.286	2.51	0.012	[1.26, 10.22]	0.089

Table S5. Model table with path estimates for the harm perception mediation model comparing harm with fairness behaviors.

Effect	Estimate	SE	z	p	CI	Std Est
c	2.821	2.400	1.18	0.24	[-1.88, 7.53]	0.043
a	24.288	2.688	9.04	< .001	[19.02, 29.56]	0.352
b	0.228	0.039	5.82	< .001	[0.15, 0.31]	0.242
ab	5.548	1.133	4.90	< .001	[3.33, 7.77]	0.085
Total effect	8.369	2.239	3.74	< .001	[3.98, 12.76]	0.128

Table S6. Model table with path estimates for the harm perception mediation model comparing harm with purity behaviors.

Effect	Estimate	SE	z	p	CI	Std Est
c	-7.466	2.241	-3.33	< .001	[-11.858, -3.074]	-0.119
a	4.724	2.658	1.78	0.076	[-0.486, 9.934]	0.071
b	0.257	0.038	6.68	< .001	[0.181, 0.332]	0.271
ab	1.213	0.706	1.72	0.086	[-0.171, 2.596]	0.019
Total effect	-6.254	2.373	-2.63	0.008	[-10.905, -1.602]	-0.099

Table S7. Model table with fit indices for the three measurement invariance models.

**Model**	**χ^2(df)**	**p**	**CFI**	**RMSEA [90% CI]**	**SRMR**	**AIC**	**ΔCFI / ΔRMSEA**
1. Configural	12.25(8)	.140	.996	.050 [.000, .093]	.018	4675.2	—
2. Metric	15.58(10)	.112	.995	.049 [.000, .088]	.022	4673.7	−.001 / −.001
3. Scalar	22.22(12)	.035	.992	.060 [.016, .099]	.024	4677.0	−.004 / +.011

Table S8. Model table of estimates for the full structural equation model examining the indirect effect of norm type on internalization through harm perceptions.

Parameter	B	SE	z	95% BCa CI	p	β
a path: Norm type → Harm perception						
Norm type (0 = social, 1 = moral)	0.194	0.024	8.24	[0.154, 0.248]	< .001	.080
b paths: Harm perception → Internalization factors						
→ Intrinsic motivation (b1)	0.305	0.045	6.78	[0.217, 0.393]	< .001	.332
→ Norm adherence (b2)	0.262	0.045	5.79	[0.174, 0.355]	< .001	.273
c′ paths: Norm type → Internalization (direct, controlling for harm perception)						
→ Intrinsic motivation (c′1)	0.009	0.106	0.09	[−0.194, 0.209]	.932	.004
→ Norm adherence (c′2)	−0.093	0.105	−0.89	[−0.302, 0.117]	.375	−.040
Indirect effects: Norm type → Harm perception → Internalization (a × b)						
→ Intrinsic motivation	0.059	0.011	5.27	[0.040, 0.085]	< .001	.064
→ Norm adherence	0.051	0.011	4.66	[0.033, 0.076]	< .001	.053
Total effects: Norm type → Internalization						
→ Intrinsic motivation	0.068	0.102	0.67	[−0.131, 0.267]	.505	.068
→ Norm adherence	−0.043	0.101	−0.42	[−0.243, 0.157]	.674	.013

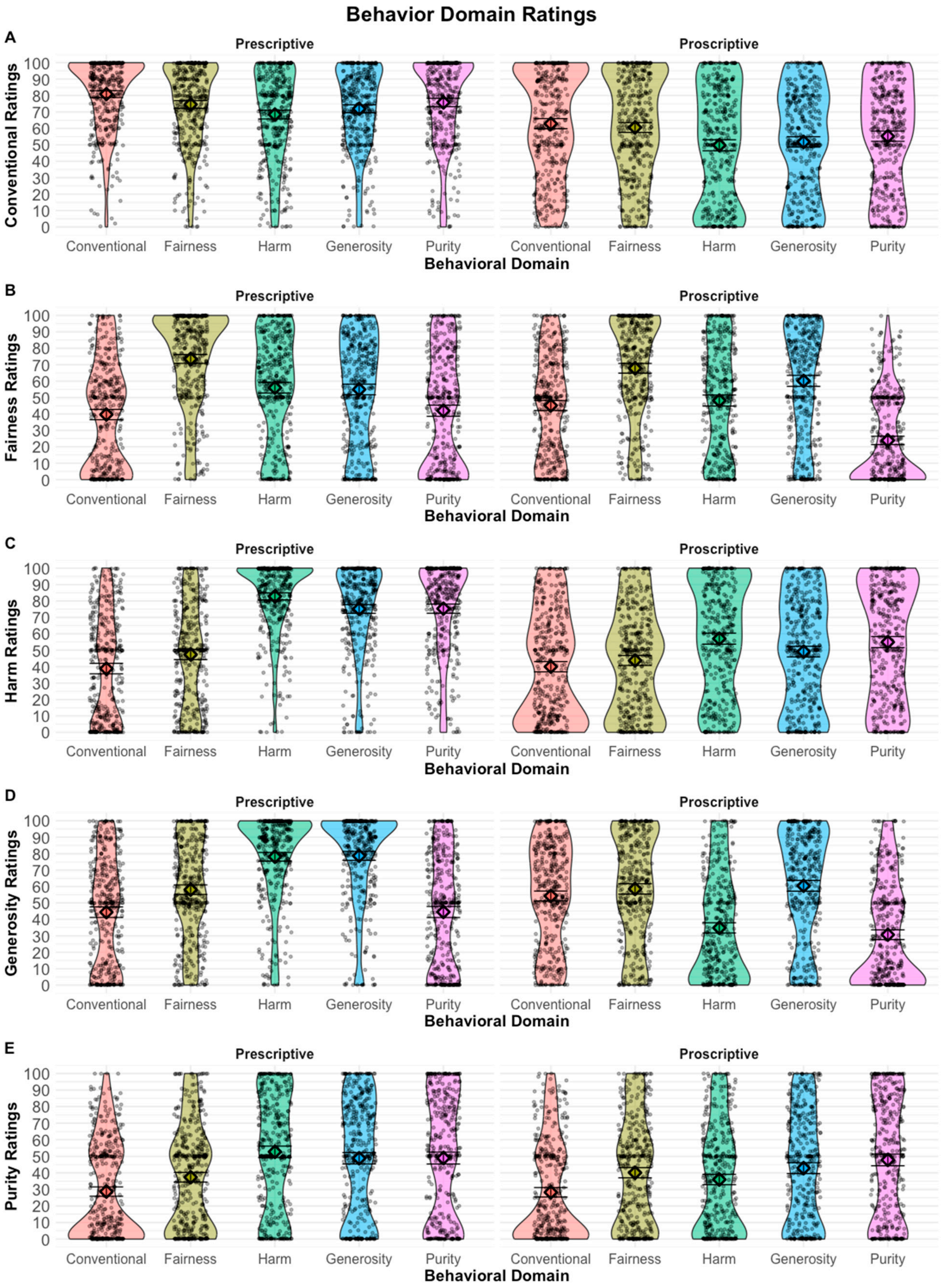

Behavior Domain Ratings
A
B
C
D
E
Prescriptive
Proscriptive
Conventional Ratings
Fairness Ratings
Harm Ratings
Generosity Ratings
Purity Ratings
0
10
20
30
40
50
60
70
80
90
100
Conventional
Fairness
Harm
Generosity
Purity
Behavioral Domain

Figure S1. Violin plots showing the means and distributions for the five behavioral dimension measures (social conventions, fairness, generosity, harm, and purity) by behavioral domain and norm valence.

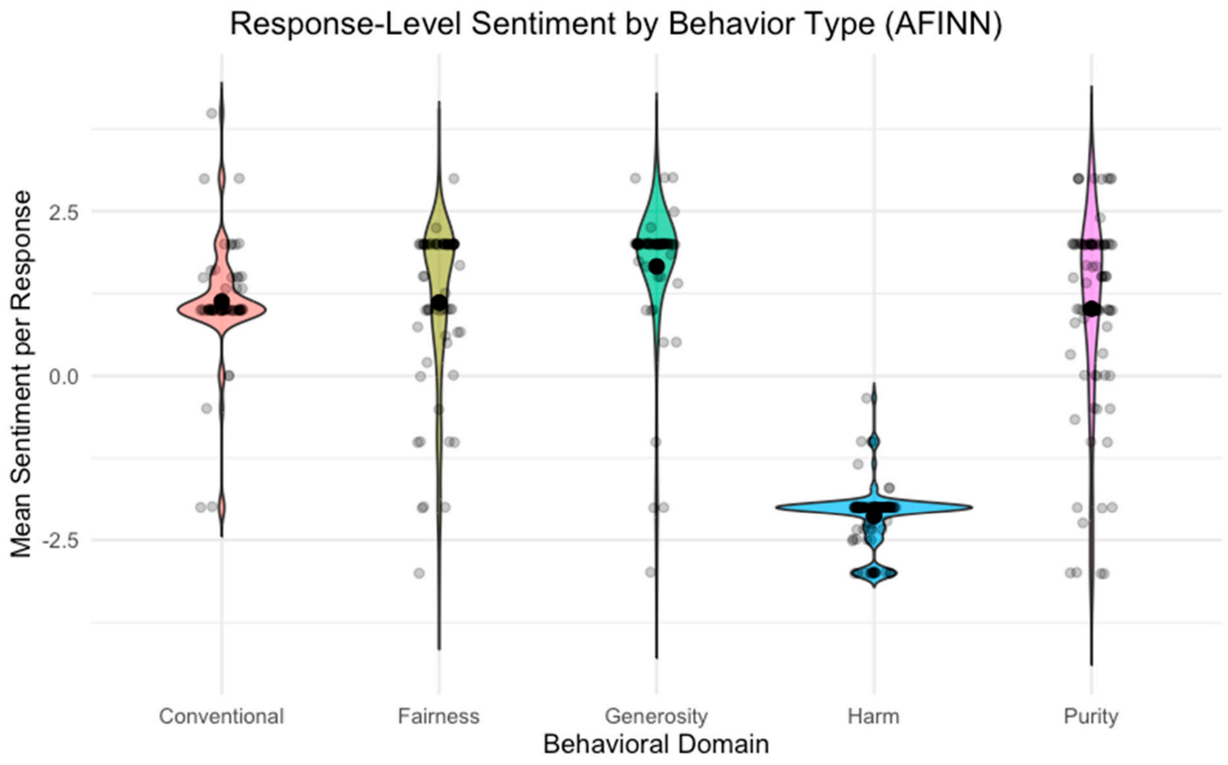

Figure S2. Violin plot showing net sentiment scores for each behavioral domain, collapsing across prescriptive and proscriptive norms.

Figure S3. Path diagram model of the full structural equation model with harm perception as a mediator.

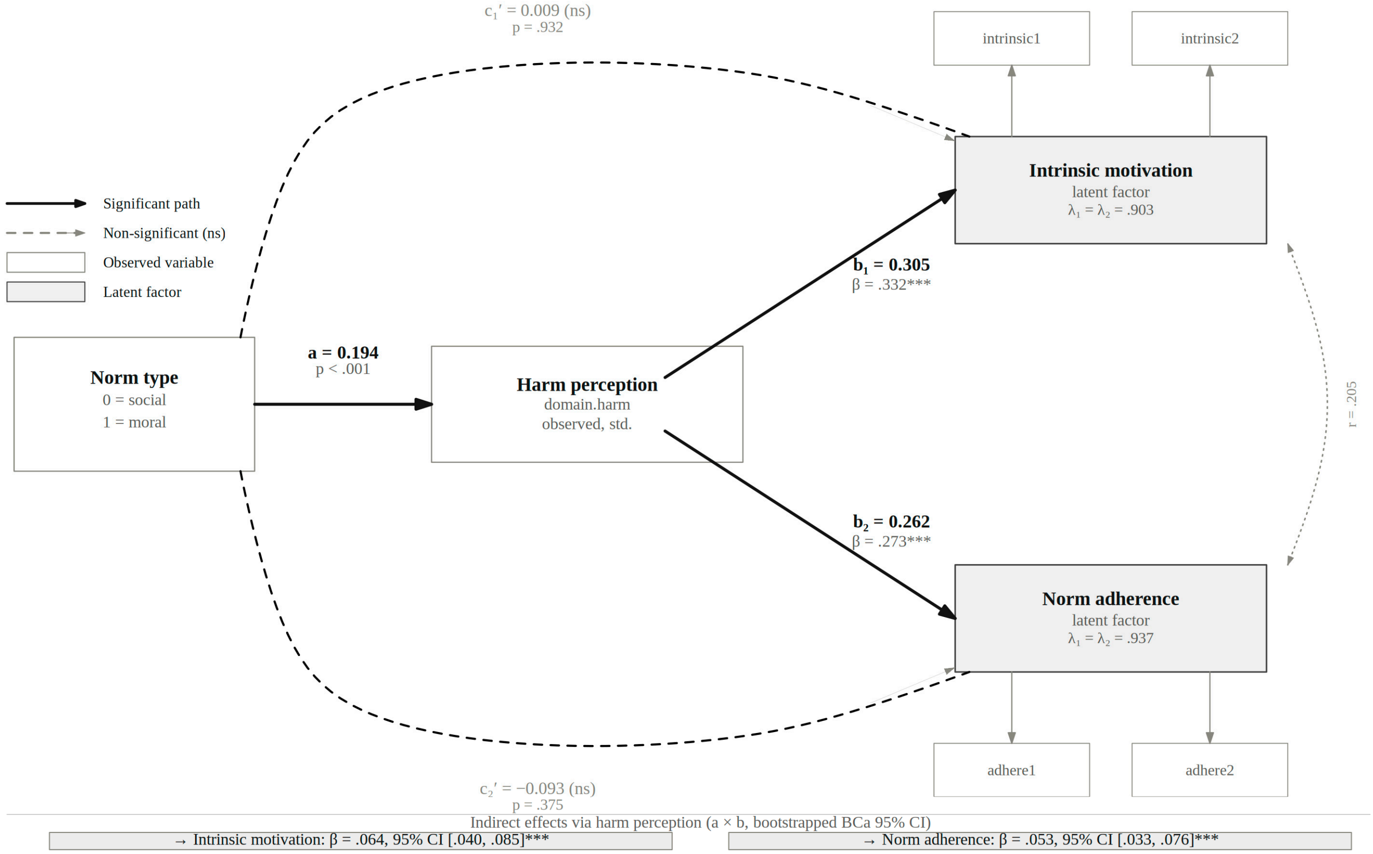

Note. β = fully standardised. B = unstandardised. ***$p < .001$.

Model fit: $\chi^2(8) = 67.10$, CFI = .962, RMSEA = .118 [.092, .144], SRMR = .068.

Standard errors and 95% CIs from 5,000 bootstrap draws (BCa correction).

Study Materials

Stimuli

<u>Conventional</u>
Prescriptive:
1. Wearing fashionable clothes
2. Shaking hands when meeting a new person
3. Being on time to meet up with friends
4. Making eye contact with the person you're talking to

Proscriptive:
1. Dressing inappropriately for a formal occasion
2. Speaking loudly in a library
3. Listening to music without headphones on public transit
4. Driving over the speed limit

<u>Fairness</u>
Prescriptive:
1. Queueing at the checkout when shopping
2. Splitting the bill based on what each person ordered
3. Equally contributing to a group project
4. Remaining neutral in a disagreement between friends

Proscriptive:
1. Cutting a line at checkout
2. Leaving dirty dishes in a shared kitchen
3. Cheating on a test or exam
4. Driving on the shoulder of the highway to get around traffic

<u>Harm</u>
Prescriptive:
1. Rescuing an injured animal
2. Buying a homeless person food
3. Consoling a grieving friend
4. Taking care of a friend or family member with a serious illness

Proscriptive:
1. Starting a false rumor about someone
2. Spanking your child
3. Intentionally tripping someone
4. Verbally harassing a store employee

<u>Generosity</u>

Prescriptive:

1. Giving money to charity
2. Donating blood
3. Volunteering at a food bank
4. Contributing to a GoFundMe to cover someone's medical bills

Proscriptive:

1. Parking in a handicap spot without a permit
2. Taking office supplies home from work for your personal use
3. Stealing a coworkers idea and presenting it as your own
4. Stockpiling essential items during an emergency

<u>Purity</u>

Prescriptive:

1. Washing your hands after using the bathroom
2. Maintaining personal hygiene
3. Choosing natural and organic products
4. Staying home when sick

Proscriptive:

1. Engaging in promiscuous sexual behavior
2. Consuming excessive amounts of junk food
3. Eating food that fell on the ground
4. Doing illicit drugs

Measures

All measures were on 100-point sliding scales.

Intrinsic Motivation

1) To what extent would you engage in the behavior because others' expect you to or because it's your personal choice? (0-Because others expect me to, 100-Because it's my personal choice)
2) To what extent would you engage in the behavior because that's what you're supposed to do or because you actually want to? (0-Because you're supposed to, 100-Because I want to)

Adherence-Avoidance Importance

<u>Proscriptive – Avoidance</u>

1) How important is it to you to avoid engaging in the behavior? (0-Not at all important, 100-Extremely important)
2) How strongly do you believe that people should avoid engaging in this behavior? (0-Not strongly at all, 100-Extremely strongly)

<u>*Prescriptive – Adherence*</u>

1) How important is it to you to engage in the behavior? (0-Not at all important, 100-Extremely important)
2) How strongly do you believe that people should engage in this behavior? (0-Not strongly at all, 100-Extremely strongly)

Reputational Sensitivity

<u>*Reputation–Public*</u>

1) Would you be more or less likely to engage in the behavior if you were around other people at the time or if your actions would be publicly known? (0-Less likely, 50-Neither less nor more likely, 100-More likely)
2) If you could engage in the behavior without anyone knowing or ever finding out, would you be more or less likely to do it? (0-Less likely, 50-Neither less nor more likely, 100-More likely)

<u>*Reputation–Common*</u>

3) How likely would you be to engage in the behavior if most people you know are engaging in it? (0-Less likely, 50-Neither less nor more likely, 100-More likely)
4) How likely would you be to engage in the behavior if most people you know are not engaging in it? (0-Less likely, 50-Neither less nor more likely, 100-More likely)

Norm Covariates

1) *Valence*: In your opinion, how emotionally positive or negative is this behavior? (0-Extremely negative, 50-Neither negative nor positive, 100-Extremely positive)
2) *Injunctive Norm Beliefs*: In your opinion, how much do other people approve or disapprove of this behavior when they are in the relevant situation? (-50-Entirely disapprove, 0-Neither disapprove nor approve, 50-Entirely approve)

3) *Descriptive Norm Beliefs*: In your opinion, how many people in your community do this behavior when they are in the relevant situation? (-50-No one, 0-Some people, 50-Everyone)
4) *Moral Judgements*: In your opinion, how moral or immoral is it to do this behavior? (-50-Extremely immoral, 0-Neither immoral nor moral, 50-Extremely moral)
5) *Punishment-Reward*: In your opinion, would the person who does this behavior get rewarded or punished by others? (-50-Definitely punished, 0-Neither punished nor rewarded, 50-Definitely rewarded)

Behavior Dimension Ratings

Participants were asked to “rate the extent to which the behavior relates to the following categories: social conventions, fairness (or unfairness), harm (or care), generosity (or selfishness), and purity (or impurity). Note that some of the categories will not be relevant for all behaviors.” (0-Not at all relevant, 100-Completely relevant)

1) Social Convention: To what extent does the behavior relate to social conventions?
2) Fairness: To what extent does the behavior relate to fairness or unfairness?
3) Harm: To what extent does the behavior relate to harm or care?
4) Purity: To what extent does the behavior relate to purity or impurity?
5) Generosity: To what extent does the behavior relate to generosity or selfishness
